# Effectiveness of mobile health interventions on physical activity management in frail older adults: a systematic review and meta-analysis

**DOI:** 10.1186/s11556-026-00411-3

**Published:** 2026-04-11

**Authors:** Yaxin Chen, Lei Wu, Jing Li, Mengyuan Yang, Xin Qi, Mei Wang, Qi Li, Yanhong Wang

**Affiliations:** 1 School of Nursing, Lanzhou University, Lanzhou, China; 2 First Hospital of Lanzhou University, Lanzhou, China

**Keywords:** Mobile health, Frail, Older adults, Physical activity, Systematic review, Meta-analysis

## Abstract

**Background:**

Mobile health plays a vital role in providing personalized treatment and management for older adults with frailty by enhancing physical activity interventions. However, there is a lack of comprehensive evidence on the efficacy of mobile health interventions in this context. This study aims to assess the impact and implementation of mobile health interventions on physical activity among adults with frailty.

**Methods:**

We conducted literature searches across multiple databases from inception to October 27, 2025, including PubMed, Web of Science, The Cochrane Library, EMBASE, CINAHL, China National Knowledge Internet (CNKI), Sinomed, WanFang Data, and Cqvip. Two reviewers independently screened and selected the publications, extracted the data, and assessed risk of bias. This study included randomized controlled trials and quasi-experimental designs. Review Manager 5.4 was used for data analysis.

**Results:**

Our systematic review identified 14 eligible studies comprising 876 participants. The modalities used in mobile health interventions included wearable devices, phone calls, internet platforms, WeChat, smartphone applications, video conferencing platform or mini programs, or a combination of technologies. The meta-analysis showed that the mobile health intervention among older adults with frailty significantly reduce frailty severity as measured by continuous indices (SMD = − 1.11, 95%CI = − 1.71, − 0.51, *P* < 0.001), and increased physical activity levels (daily step count (SMD = 0.89, 95%CI = 0.41, 1.38, *P* < 0.001), and Moderate-to-Vigorous Physical Activity (MVPA)(SMD = 0.40, 95%CI = 0.10, 0.70, *P* < 0.05)), physical function outcomes (gait speed (SMD = 1.25, 95%CI = 0.91, 1.59, *P* < 0.05), TUG (MD = − 2.21, 95%CI = − 3.63, − 0.80, *P* = 0.002), and Short Physical Performance Battery (SPPB) (MD = 1.32, 95%CI = 0.73, 1.92, *P* < 0.05)), and mental domain of quality of life (MCS) (MD = 3.34, 95%CI = 0.16, 6.52, *P* = 0.04).

**Conclusions:**

Physical activity interventions for older adults with frailty using mobile health technologies are beneficial for managing frailty, improving physical function, and increasing physical activity levels effectively. High-quality research is warranted to bolster the evidence in these fields in the future.

**Supplementary Information:**

The online version contains supplementary material available at 10.1186/s11556-026-00411-3.

## Introduction

With the intensification of global population ageing, frailty has emerged as a major public health issue that urgently needs to be addressed, as it represents a prominent health risk among older adults. Frailty is defined as a non-specific clinical state in older adults characterized by reduced physiological reserves, increased vulnerability of the organism, and diminished ability to cope with stressors [1]. The prevalence of frailty among older adults remains high. A pooled analysis of 1.76 million older adults from 62 countries reported a frailty prevalence of 12%, with the prevalence of pre-frailty reaching as high as 30% [2]. Frailty substantially impairs physical functioning, exacerbates dependence in daily life [3], and increases the risk of falls, fractures, disability, and even death in older adults [4]. Importantly, frailty is increasingly regarded as a dynamic and, to some extent, reversible condition [5]. However, the likelihood of reversibility depends on the stage (e.g., pre-frailty vs. established frailty), the affected domains (physical, cognitive, psychosocial), and the intensity and type of intervention. This view is consistent with contemporary frailty models: the physical phenotype model conceptualizes frailty as a clinical syndrome characterized by weakness, slowness, exhaustion, weight loss and low activity [6], whereas the deficit accumulation model views frailty as the cumulative burden of health deficits captured by a frailty index [7]. In addition, multidimensional models incorporate physical, psychological and social deficits to better reflect the heterogeneity of frailty trajectories [8, 9]. All three models highlight physical inactivity as a core modifiable risk factor, making physical activity intervention a cornerstone of frailty management [10, 11].

Physical activity are now core recommendations in clinical practice guidelines for frailty management [11–13]. Physical activity refers to any bodily movement produced by skeletal muscles that results in energy expenditure [14]. Conceptually, physical activity can be accumulated across domains such as work/occupation, transportation, household activities, and lei-sure-time activity [15]. For frail older adults, interventions often focus on safe leisure-time exercise and on integrating light-to-moderate activity into daily routines (e.g., walking and functional tasks), with plausible benefits via neuromuscular adaptations (improved strength, balance, and gait), metabolic benefits (improved insulin sensitivity and mitochondrial function), and psychosocial pathways (enhanced mood, self-efficacy, and social engagement) [11, 16]. Conversely, excessive sedentary behaviour (e.g., prolonged sitting or reclining with very low energy expenditure) may exacerbate muscle disuse, cardiometabolic risk, and functional decline, thereby contributing to frailty onset and progression [17]. Importantly, sedentary behaviour is conceptually distinct from physical inactivity: a person may meet moderate-to-vigorous physical activity recommendations yet still accumulate high sedentary time [17, 18]. For frail older adults, both increasing overall activity and interrupting prolonged sedentary bouts may therefore be meaningful targets for intervention.

Traditional modes of physical activity intervention, such as face-to-face programmes delivered in outpatient clinics or community centres, are often constrained by limitations in time, space, and human resources [19, 20]. These approaches tend to have limited coverage, a low degree of individualization, and poor patient adherence, and thus fail to meet the long-term, continuous physical activity management needs of frail older adults [19]. Moreover, even when supervised programmes improve physical activity during the intervention period, activity levels frequently decline after programme cessation, indicating that the central challenge is the sustained integration of active behaviours over time rather than short-term participation alone [21]. Therefore, innovative approaches are needed to support long-term behaviour maintenance while reducing structural barriers to access.

In recent years, with the rapid development of information and communication technologies (ICT), mobile health (mHealth) interventions have been gradually introduced into older populations [22]. mHealth is a key part of digital health and involves utilizing mobile devices like phones and wireless tools to support medical and public health activities. In physical activity management, mHealth can enable remote monitoring [23, 24], individualized feedback [25], behavioural prompts and goal-setting support [26], and social interaction or peer support [27]. By embedding behaviour change techniques (e.g., self-monitoring, feedback, graded goals, reinforcement) into daily life, technology-mediated interventions may help overcome temporal and spatial constraints and provide continuous support that is difficult to sustain with purely in-person models, making them a plausible strategy for frailty prevention and management. In addition, because these interventions can be delivered remotely, mHealth interventions may offer a scalable and potentially cost-effective approach to physical activity management. This public health potential may be particularly relevant in ageing societies, where sustainable frailty management requires models that can extend service reach without proportionally increasing workforce and infrastructure demands.

However, evidence for mHealth interventions specifically designed to manage physical activity in frail older adults remains fragmented. Prior reviews have examined mHealth apps for reducing sedentary time and increasing physical activity in older adults in general [22, 26], and broader telehealth interventions targeting frailty or physical function [28–30], yet they often combined heterogeneous digital modalities, included largely robust older adults, or did not focus on physical activity management as the core therapeutic target. In addition, few reviews have quantitatively synthesised both frailty-related outcomes and objectively measured physical activity outcomes while also considering implementation indicators (e.g., adherence, engagement, safety), which are critical for translating digital interventions into practice [31, 32]. Therefore, an updated synthesis focusing on frail populations and mHealth-supported physical activity management is warranted.

This systematic review and meta-analysis of RCTs and quasi-experimental studies aims to: (1) systematically synthesize the details of all available mHealth interventions of physical activity management in frail older adults, (2) preliminarily evaluate the overall efficacy of these interventions, and (3) evaluate implementation outcomes linked with the delivery of mHealth interventions in this population.

## Methods

This study followed the Preferred Reporting Items for Systematic Reviews and Meta-Analyses (PRISMA) guidelines [33], and the protocol was registered with PROSPERO (CRD420251249011).

### Search strategy

A systematic literature search was conducted using PubMed, EMBASE, Web of Science, the Cochrane Library, CINAHL, Chinese National Knowledge Infrastructure (CNKI), Wanfang databases and Cqvip from inception to October 27, 2025. We developed the search terms using a combination of subject terms and accessible terms. The search strategy, detailed in Supplementary Table 1, encompassed keywords related to mHealth interventions (e.g., wearable technology, messaging, mobile applications, etc.), frail older adults (e.g., frailty, frail etc.), and physical activity (e.g., activity, exercise, walking, sedentary behavior etc.).

### Inclusion and exclusion criteria

Using the PICOS model as a framework, the inclusion criteria were (1) type of study: RCTs or quasi-experimental studies; (2) participants: studies investigating participants with a mean age of 60 + years or older who were identified as frail or pre-frail using validated frailty assessment instruments (e.g., Fried Frailty Phenotype (FFP), Fried Frailty Index (FFI), FRAIL Scale, etc.); (3) intervention group: physical activity interventions employing mobile health techniques—such as pedometers, bracelets, accelerometers, Email, Short Message Service, cellphones, apps, mini programs, WeChat, or internet platforms—as the intervention; (4) control group: usual care or alternative physical activity interventions without an mobile health component; (5) outcome indicators: any measures related to frailty, physical activity, physical function, quality of life, or implementation of the intervention. The exclusion criteria were duplicate publications or literature with incomplete reported data.

### Data selection and extraction

The study selection process was managed using EndNote X9. After duplicate records were removed, two reviewers independently screened the titles and abstracts of all retrieved records. Full-text articles of potentially eligible studies were then independently assessed by the same two reviewers according to the predefined inclusion and exclusion criteria. Any discrepancies were first resolved through discussion between the two reviewers, and if consensus could not be reached, a third reviewer was consulted for adjudication.

Data extraction was independently performed by two reviewers using a prespecified standardized electronic data extraction form. The form was developed a priori based on the review objectives and the PICOS framework. It included the following items: author, year of publication, country, study setting, participant characteristics (including population, inclusion and exclusion criteria, sample size, and age), intervention and comparator details (including content, type of mobile health technology, and intervention duration), and outcome indicators.

No formal inter-rater agreement statistic (e.g., Cohen’s kappa coefficient) was calculated in this review. In addition, no formal pilot testing of the data extraction form was conducted before full data extraction. However, the form was used in a standardized manner by two independent reviewers throughout the review process to enhance consistency and transparency.

### Outcome domains and measurement approaches

The effectiveness outcomes of interest included: (1)frailty, assessed using validated frailty instruments reported by the original studies, such as the FFP [6], Study of Osteoporotic Fracture (SOF) [34], FFI [35], Tilburg Frailty Indicator (TFI) [9], Clinical Frailty Scale (CFS) [7], FRAIL Scale [36], and Groninger Frailty Indicator (GFI) [37]; (2) physical activity, including daily step count, sedentary behavior, Moderate-to-Vigorous Physical Activity (MVPA), and exercise self-efficacy; (3) physical function, including handgrip strength, gait speed, Timed Up and Go test (TUG) [38], and Short Physical Performance Battery (SPPB) [39]; (4) exercise self-efficacy, referring to participants’ confidence in their ability to initiate and maintain exercise or physical activity behaviors, as assessed by validated self-efficacy-related instruments reported in the original studies; (5) quality of life (QoL), assessed using validated instruments such as the EuroQol 5-dimensional questionnaire (EQ-5D), Visual Analog Scale of the EuroQol 5-dimensional questionnaire (EQ-5D VAS) [40] and 12-Item Short-Form Health-Survey (SF-12), which is divided into physical component summary (PCS) and mental component summary (MCS) [41].

Implementation-related outcomes included reach, feasibility, adherence, and safety, as reported by the original studies.

### Quality assessment

For RCTs, the risk of bias was assessed by two independent reviewers using the Cochrane Risk of Bias Tool (version 5.1.0) [42]. Seven domains were evaluated: selection bias (random sequence generation, allocation concealment), implementation bias (blinding of participants and personnel), measurement bias (blinding of outcome assessment), follow-up bias (incomplete outcome data), reporting bias (selective reporting), and other sources of bias. Each domain was judged as “low risk,” “unclear risk,” or “high risk.” Disagreements were resolved through discussion with a third reviewer.

For quasi-experimental studies, we assessed the risk of bias using the appraisal tool from the Joanna Briggs Institute (JBI) Centre for Evidence-Based Healthcare, Australia [43]. This tool consists of nine appraisal items, and reviewers are required to judge each item as “Yes,” “No,” “Unclear,” or “Not Applicable.” Disagreements were resolved through discussion with a third reviewer.

### Data analysis

Meta-analysis was performed using Review Manager 5.4 statistical software. Studies were pooled only when two or more studies assessed the same underlying outcome con-struct and reported sufficiently comparable data. To minimize methodological heteroge-neity arising from differences in outcome instruments, meta-analysis was undertaken only when outcomes were considered comparable in terms of construct, measurement, and reporting. If studies were not suitable for meta-analysis because of substantial differences in outcome definitions, measurement protocols, or baseline comparability, their findings were summarized narratively. For continuous variables, the mean difference (MD) was used when the same outcome was measured using the same instrument and unit, whereas the standardized mean difference (SMD) was used when the same construct was measured using different scales or units. Dichotomous variables were evaluated using relative risk (RR). Heterogeneity was quantified using the I² statistic. An I² value < 50% was considered to indicate low heterogeneity, and a fixed-effects model was used; otherwise, a ran-dom-effects model was applied [44]. If sufficient studies were available (three or more), subgroup analyses were conducted. Sensitivity analyses were performed to test the robustness of the results by excluding studies with a high risk of bias [45].

## Results

### Study selection

**Fig. 1:**
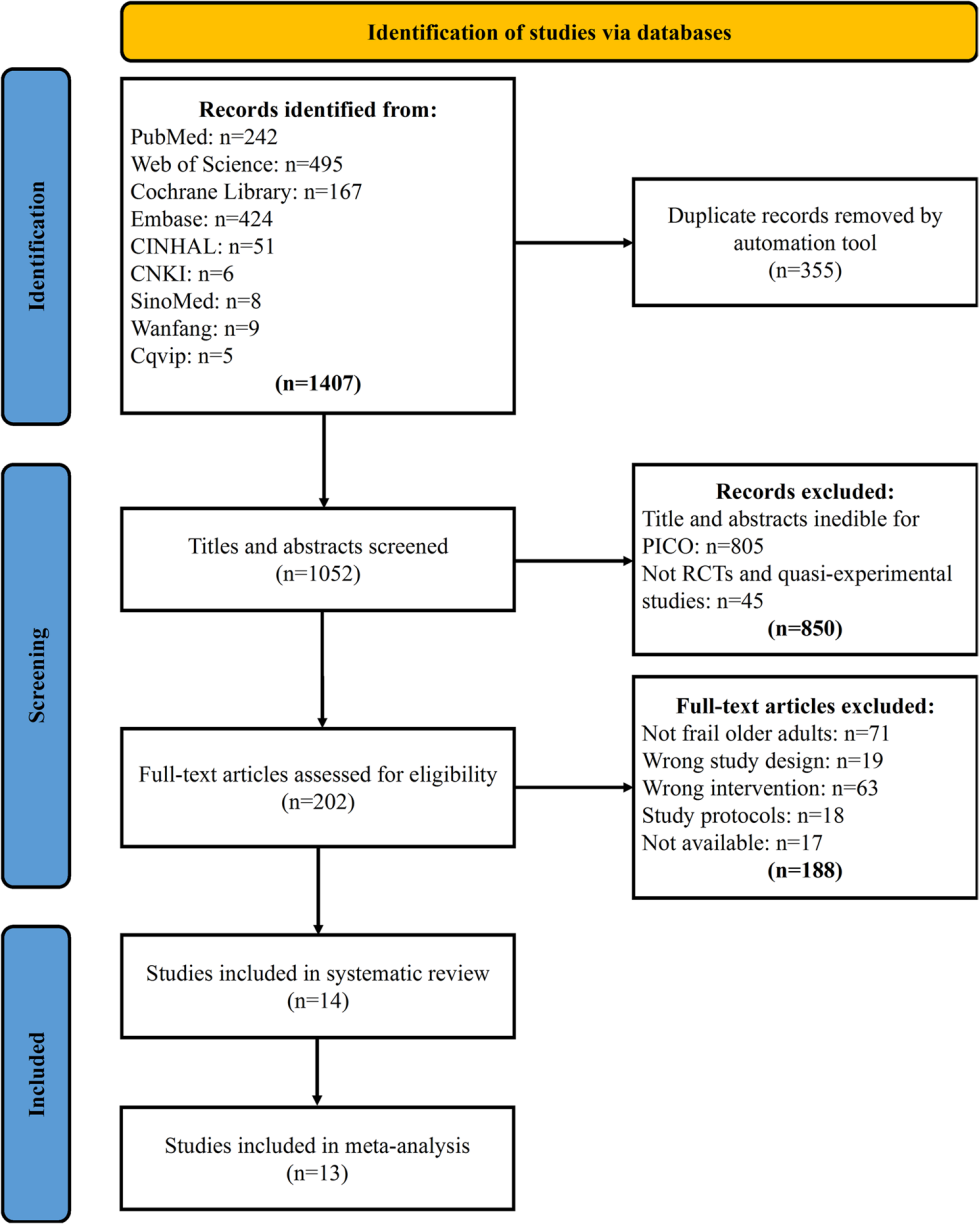
Flow diagram of literature search

A total of 1407 records were identified in the initial search, and after deduplication followed by the title, abstract, and full-text screening, 14 studies were included in this systematic review, of which 13 were included in the meta-analysis (Fig. 1). One study [46] was excluded from the meta-analysis due to the lack of reported means and standard deviations required for effect size calculation. Among the 13 studies, 2 were quasi-experimental studies [47, 48], and the remaining 11 were RCTs.

### Characteristics of included studies

Table 1 summarizes the characteristics of the included studies. The included studies were published between 2017 and 2025, with the majority published in 2025 (6 studies, 42.9%). The studies were conducted in various regions, including China (*n* = 9), the United Kingdom (*n* = 1), Netherlands (*n* = 1), France (*n* = 1), Spain (*n* = 1), and South Korea (*n* = 1). Sample sizes ranged from 16 to 134 participants, with a total of 876 participants across all studies.

**Table 1:** Characteristics of the included studies

Study/ Setting	Population/ Sample size	Age*	Mobile health technology and key features	Duration Time	Control	Outcomes
Bailey et al. (2024) [49]; Community, England	Pre-frai (CFS); *n* = 60 (In = 30, Cn = 30)	74.0 ± 6.0	①Wearable devices: provides real-time inactivity feedback and alerts to promote self-monitoring of sedentary behavior; tracked steps and activity data; ②Video conferencing platform or phone calls: enabled remote one-on-one health coaching; ③APP: facilitated ongoing peer interaction to enhance support and motivation.	24 weeks	Usual care	Sedentary behavior; Physical Function (hand grip strength, SPPB); ADL; Quality of Life and Mental Health
Dekker-van et al. (2017) [50]; Residential care, Netherlands	Pre-frail (SF-36 Physical Functioning Scale ≤ 60 or GFI = 4); *n* = 36 (In = 15, Cn = 21)	In: 69.2 ± 3.8; Cn: 70.9 ± 3.5	Internet platforms: provided video exercise guidance, with therapists able to remotely adjust prescriptions and give feedback.	12 weeks	Usual care	Use (frequency/duration); User experience (SUS); Quality of life (SF-12 PCS, MCS); Health status (EQ-5D-3 L, EQ-VAS)
Kwan et al. (2020) [51]; Community, Hong Kong, China	Pre-frail/frail (FFI ≥ 1) and MCI; *n* = 33 (In = 16, Cn = 17)	71.0(66.5, 75.5)	APP: automatically tracked walking behaviors (steps, speed, time, etc.) and delivered personalized message notifications based on the activity data.	12 weeks	Conventional behavior change intervention (health education and brisk walking training)	Frailty (FFI); Cognitive function (MoCA); Walking-related indicators (total walking time, brisk walking time (> 100 steps/min), daily step count, 1-minute peak cadence); MVPA; Hand-grip strength; 6MWT; Physical Activity Scale for the Elderly
Lai et al. (2025) [52]; Community, China	Frail (FFP ≥ 3) and MCI; *n* = 72 (In = 36, Cn = 36)	75.66 ± 7.9	WeChat: provided instructional videos; conducted clock-in via WeChat groups; and monitored participants’ training compliance in real time.	16 weeks	Routine health education	Frailty (TFI); Cognitive function (MoCA); Balance and gait function (Perfor-mance-Oriented Mobility Assessment); TUG; Dual-task cognitive load (Smart pen combined with writing task and sound recognition task, and dual-task cost (DTC) was calculated)
Lee et al. (2025) [53]; Community, Hong Kong, China	Pre-frail/frail (FRAIL scale ≥ 1); *n* = 38 (In = 19, Cn = 19)	In: 69.0 ± 8.18; Cn: 74.28 ± 9.82	APP: provided a library of outdoor exercise facilities, location-based search for outdoor exercise, guidance on exercise risk management, step-by-step audio instructions, demonstration videos, and professional advice from physiotherapists and occupational therapists.	4 weeks	Health education workshops, including exercise experiential sessions tailored	Self-reported physical activity level (Rapid Assessment of Physical Activity Scale); Utilization of outdoor exercise facilities (Self-developed questionnaire); MVPA; Exercise self-efficacy (SEE-C); Mental Well-being (Chinese Short Warwick-Edinburgh Mental Well-Being Scale).
Li et al. (2025) [54]; Community, China	Pre-frail (1 ≥ FFP ≥ 2); *n* = 134 (In = 67, Cn = 67)	In: 69.3 ± 5.1; Cn: 69.2 ± 6.3	①Wearable devices: monitored steps, activity intensity, and sedentary time. ②APP: provided personalized exercise plans and video guidance. ③Remote management platforms: enabled healthcare professionals to upload and design personalized exercise plans, allowed them to remotely and real-time access participants’ exercise data, and supported them in sending reminders and motivational messages to interact with participants.	24 weeks	Routine health education	Frailty (FFP); 30-s chair stand; Handgrip strength, TUG; Gait speed; Balance tests; Body composition (BMI, fat mass, fat-free mass); Bone mineral density; Physical activity; Sedentary behavior
Lin et al. (2022) [55]; Long-term care facility, Taiwan, China	Pre-frail/frail (SOF ≥ 1); *n* = 16 (In = 8, Cn = 8)	In: 86.25 ± 5.15; Cn: 82.5 ± 6.55	①Wearable devices: collected exercise data and physiological data; ②Remote management platforms: automatically generated personalized exercise prescriptions and dynamically adjusted training plans based on user performance; it also provided remote health monitoring and abnormal warning functions.	12 weeks	Regular physical training sessions provided by the long-term care facility	Frailty (SOF); Body Composition (BMI, Skeletal Muscle Index); Muscle Strength (grip strength, elbow flexor/extensor strength, knee flexor/extensor strength, hip flexor strength); Physical Function (TUG, SPPB, 4-meter gait speed); Health-related Quality of Life (SARC-F scale, GDS-5, Short Portable Mental Status Questionnaire, EQ-5D-3 L, Katz ADL)
Liu et al. (2021) [56]; Community, Hong Kong, China	Pre-frail/frail (FFI ≥ 1); *n* = 40 (In = 22, Cn = 18)	In: 75.9 ± 6.74; Cn: 72.1 ± 3.7	Wearable devices: monitored exercise data and included functions such as goal setting, feedback, and rewards.	14 weeks	Received physical training and health education talks (without the wearable devices).	Frailty (FFI); MVPA; Step count; Step cadence; Physical Function (TUG, 30 s Chair Stand Test, 2MWT); Exercise self-efficacy (SEE-C); Motivation to engage in physical activity (Behavioral Regulation in Exercise Questionnaire-2)
Piau et al. (2021) [46]; Community, France	Pre-frail/frail (FFP ≥ 1); *n* = 35 (In = 25, Cn = 10)	In: 79.3 ± 5.9; Cn: 77.8 ± 5.9	Wearable devices: monitored walking-related indicators in real time, and provided activity data feedback.	12 weeks	Usual care	Frailty (FFP); Physical Function (SPPB); ADL; Health-related quality of life (SF-36, EQ-5D-3 L)
Valdés-Aragonés et al. (2024) [57]; Geriatrics outpatient clinics, Spain	Pre-frail/frail (FFP ≥ 1); *n* = 90 (In = 46, Cn = 44)	In: 82.11 ± 5.42; Cn: 82.56 ± 6.43	①Sensors kit: monitored key functional indicators such as gait speed, lower limb strength, and body weight; ②APP: sent test reminders and encouraged patients to complete functional assessments regularly; ③Remote management platforms: medical staff were able to remotely access data, receive system-generated deterioration alerts, and adjust treatment plans.	24 weeks	Usual care	Frailty (FFP, FTS-5); Health-related quality of life (EQ-5D-5 L); Healthcare resource use (emergency visits, hospital admissions, falls, etc.)
Lee, Kyeongjin. (2025) [58]; Residential care, Korea	Pre-frail (1 ≥ FFP ≥ 2); *n* = 60 (In = 30, Cn = 30)	In: 84.90 ± 2.85; Cn: 84.81 ± 2.71	Video conferencing platform: provided real-time exercise guidance and remote supervision.	8 weeks	Received an informational booklet on exercise and a single educational session on fall prevention and physical activity	Balance ability (TUG, Berg Balance Scale, Activities-specific Balance Confidence Scale); Lower-limb strength (Five Times Sit-to-Stand Test, 30 s Chair Stand Test); Gait ability (Dynamic Gait Index, 10MWT); Fall efficacy (MFES)
Zhang et al. (2023) [59]; Community, China	Pre-frail/frail (FFP ≥ 1); *n* = 116 (In = 58, Cn = 58)	In: 67.7 ± 4.9; Cn: 68.8 ± 4.8	WeChat mini-program: delivered exercise video courses and provided scheduled reminders, automatic check-ins, and progress tracking.	12 weeks	Usual community center services	Frailty (FFP); Exercise adherence
Li et al. (2025) [47]; Community, Taiwan, China	Pre-frail/frail (FFP ≥ 1); *n* = 81 (In = 43, Cn = 38)	In: 74.95 ± 7.31; Cn: 78.61 ± 7.49	APP: pushed exercise and nutrition guidance, customized personalized physical activity and nutrition tasks, and provided remote consultation and guidance.	24 weeks	Usual community center services	Frailty (FFP); Health-related quality of life (SF-12); Fall efficacy (Short Falls Efficacy Scale International)); Pain intensity (VAS); Daily activities ((IADL); Behavior change stages (Behavioral Change Stages and Satisfaction Questionnaire)
Xue et al. (2025) [48]; Residential care, China	Pre-frail/frail (FFP ≥ 1) and MCI; *n* = 65 (In = 31, Cn = 34)	In: 73.87 ± 3.72; Cn: 73.44 ± 4.26	①App: provided video demonstrations and guided participants to complete training, offerd health educatio, and tracks progress. ②WeChat: used for communication, encouragement, and weekly training supervision. ③Video Call: for online evaluation of training adherence and correction.	12 weeks	Routine health education	Frailty (FFP); Cognitive function (MoCA); Physical Function (SPPB, 4-meter gait speed, and grip strength); Fall efficacy (Modified Falls Efficacy Scale); Fall history

*[(mean ± SD/median (IQR)]

*CFS* Clinical Frailty Scale, *In* Intervention group, *Cn* Control groupm, *SPPB* Short Physical Performance Battery, *ADL* Activity of Daily Living, *GFI* Groninger Frailty Indicator, *SUS* System Usability Scale, *SF-12* 12-Item Short-Form Health-Survey, *SF-12 PCS, MCS* SF-12 Phys-ical Component Scale, Mental Component Scale, *EQ-5D-3 L* EuroQol Five-Dimensions Three-Level questionnaire, *EQ-VAS* EuroQol Visual An-alogue Scale, *FFI* Fried Frailty Index, *MCI* Mild cognitive impairment, *MoCA* Montreal Cognitive Assessment, *MVPA* Moderate-to-vigorous physical activity, *MWT* Minute walk test, *TFI* Tilburg Frailty Indicator, *TUG* Timed Up and Go Test, *SEE-C* Chinese ver-sion of the Exercise Self-Efficacy Scale, *BMI* Body Mass Index, *SOF* Study of Osteoporotic Fractures, *SARC-F scale* Strength, Assistance walking, Rise from a chair, Climb stairs, Falls, *GDS-5* Geriatric Depression Scale-5, *SF-36* 36-Item Short Form Health Survey, *FTS-5* Frailty Trait Scale-5 items, *VAS* Visual Analogue Scale, *IADL* Instrumental Activities of Daily Living

The average ages of participants ranged from 66.0 to 86.25 years. All studies targeted pre-frail or frail older adults. Among the 14 included studies, frailty was assessed using the CFS [49], GFI [50], FRAIL scale [53], SOF [55] in one study each, and the FFI in two studies [51, 56]. The remaining eight studies employed the FFP. Three studies included participants with mild cognitive impairment (MCI) in addition to frailty [48, 51, 52], but all participants in these studies still met predefined frailty-related eligibility criteria.

### Mobile health interventions

#### Modalities of mHealth

This review included studies that employed four primary mHealth delivery modalities (with some studies using multi-modal combined interventions): (1) Apps (*n* = 7) [47–49, 51, 53, 54, 57]: Health management tools delivered through smartphones or tablets. (2) Online communication software (*n* = 5) [48, 52, 55, 58, 59]: Including WeChat, videoconferencing platforms, and telephone calls. (3) Remote management platforms (*n* = 4) [50, 54, 55, 57]: Existing mature platforms or independently developed systems leveraging internet-based technologies. (4) Wearable devices (*n* = 5) [46, 49, 54–56]: Such as smart wristbands, primarily used to objectively collect physical activity-related data like step counts.

#### Intervention contents

The components of mHealth interventions for managing physical activity in frail older adults encompass five categories: (1) Monitoring: Utilizing wearable devices [49, 54–57], apps [51], and remote management platforms [54, 57] to continuously collect participants’ activity data (e.g., step count, sedentary time) and physiological indicators. The system can automatically detect abnormalities and trigger alerts. (2) Personalized exercise guidance: Through apps [47, 53, 54], remote management platforms [50, 55, 57], and online communication software [49], personalized exercise plans are developed based on the participant’s age, frailty level, and monitoring data, with dynamic adjustments made to the training program based on participants’ performance. (3) Health education: Delivered via apps [47, 48, 53, 54], online communication software [59], and remote management platforms [54], health education content such as exercise knowledge, nutritional recommendations, and risk prevention is provided through short videos, image-text push notifications, and information alerts. (4) Remote support and supervision: Through online communication software [48, 49, 52, 57] and remote management platforms [50, 54], remote exercise guidance and movement corrections are offered. (5) Feedback and behavioral promotion (reminders, check-ins, goals/rewards): Self-monitoring and long-term adherence are promoted through personalized message reminders based on activity data, scheduled notifications, automatic check-ins, progress tracking, as well as goal setting and reward mechanisms [49, 51, 52, 54–57, 59].

### Quality assessment

All of the 11 included RCTs demonstrated a low risk of bias in random sequence generation, attrition bias, and reporting bias. However, most RCTs (90%, *n* = 10) exhibited a high risk of bias in participant and personnel blinding (Supplementary Figs. 1 and 2). For quasi-experimental studies, two studies received a “Yes” rating on all 9 items, indicating good overall methodological quality (Supplementary Table 2).

### Measurement of outcomes

#### Frailty

**Fig. 2:**
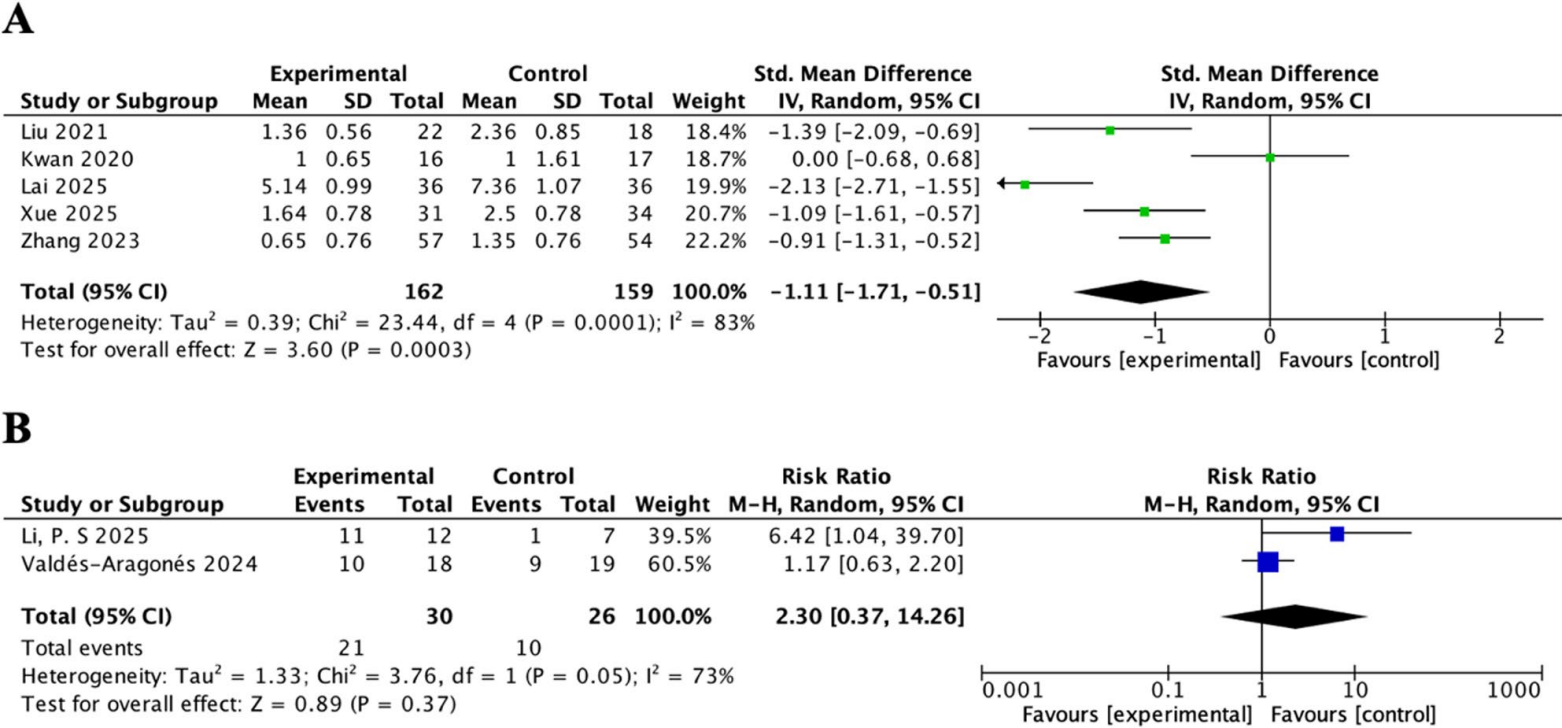
Forest plots for (**A**) Fraity (continuous frailty scores); (**B**) Fraity (dichotomous frailty status)

Because frailty outcomes were reported heterogeneously across studies, we performed separate meta-analyses according to outcome type. Continuous frailty measures referred to numerical scores derived from validated frailty instruments, which reflect the severity of frailty on a scale. Categorical frailty measures referred to frailty status transitions, such as improvement from frail to prefrail/robust or from prefrail to robust, which were treated as dichotomous outcomes. Five studies reported frailty as a continuous outcome [48, 51, 52, 56, 59]. Pooled analysis showed that, compared to control groups, mHealth interventions demonstrated a statistically significant effects in reducing frailty (SMD = − 1.11, 95%CI = − 1.71, − 0.51, *P* < 0.001, Cochran’s Q = 23.44, *p* < 0.001, I² = 83%, random-effects model). Despite significantly high heterogeneity, all five studies reported a significant decrease in frailty scores after the intervention (Fig. 2A).

Three studies reported frailty as a dichotomous outcome. Among them, two trials reported the improvement rate of frailty (transition from frail to a prefrail or robust status) and were pooled in a meta-analysis [47, 57]. The combined results showed no statistically significant difference in frailty improvement between the mHealth and control groups (RR = 2.30, 95% CI = 0.37, 14.26, *P* = 0.37, Cochran’s Q = 3.76, *p* = 0.05, I² = 73%, random-effects model) (Fig. 2B). One additional study [54] focused on pre-frail older adults and reported the improvement rate of pre-frailty to robust status as the outcome. The trial showed that mHealth interventions markedly increased the improvement rate compared with usual care (*p* < 0.001).

#### Physical activity

##### Daily step count

**Fig. 3:**
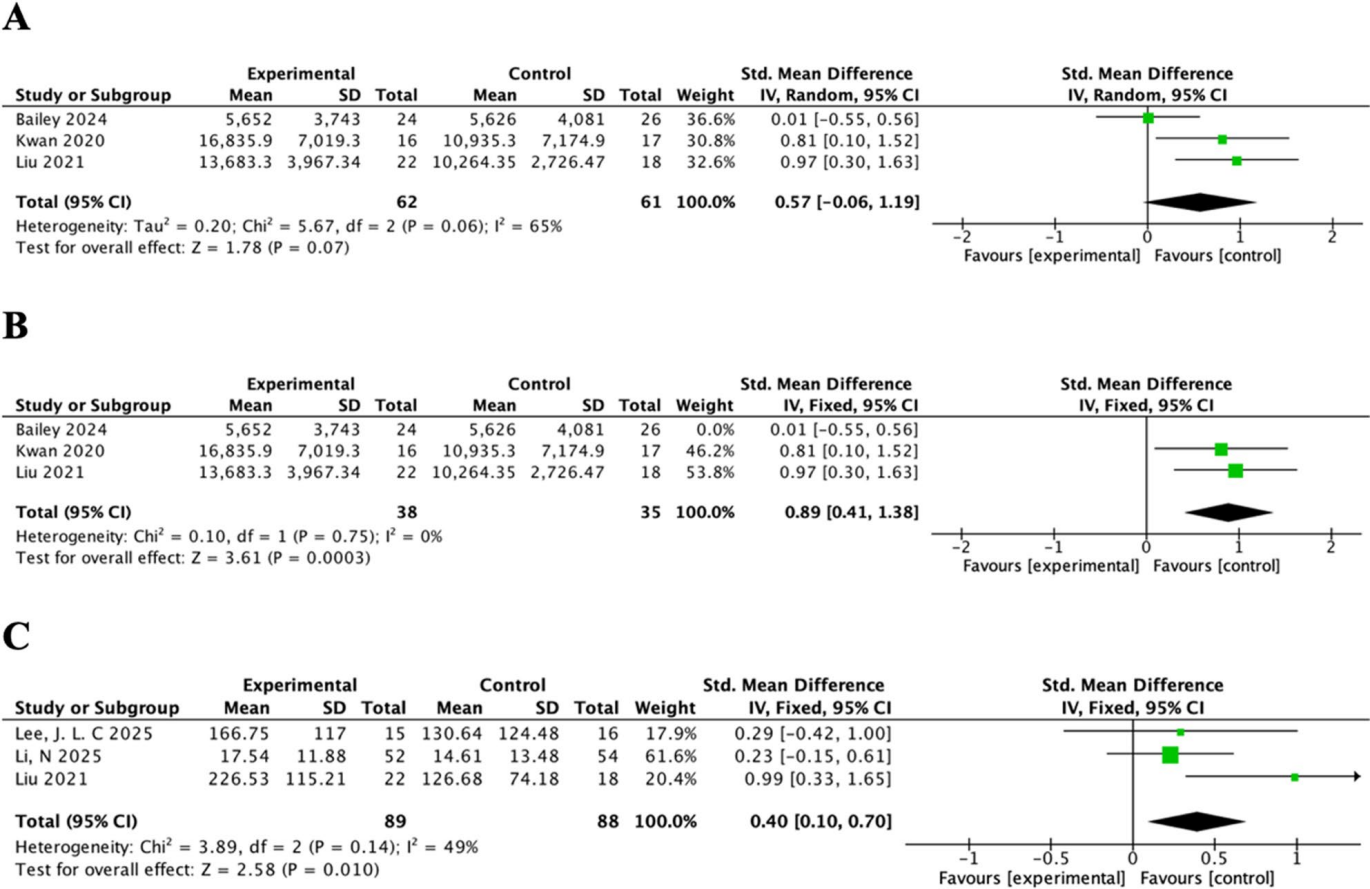
Forest plots for (**A**) daily step count; (**B**) daily step count (sensitivity analysis); (**C**) Moderate-to-Vigorous Physical Activity (MVPA)

Three studies assessed daily step count [49, 51, 56]. Compared with the control group, the intervention demonstrated no statistically significant effect on increasing daily step count (SMD = 0.57, 95%CI = − 0.06, 1.19, *P* = 0.07, Cochran’s Q = 5.67, *p* = 0.06, I² = 65%, random-effects model) (Fig. 3A). Following the exclusion of the study by Bailey, D. P et al. in a sensitivity analysis [49], heterogeneity was eliminated. Using a fixed-effect model on the remaining two studies, the pooled analysis showed a significant effectiveness on increasing daily step count (SMD = 0.89, 95%CI = 0.41, 1.38, *P* < 0.001, Cochran’s Q = 0.10, *p* = 0.75, I² = 0%) (Fig. 3B).

##### Sedentary behavior

Two studies reported daily sedentary time as an outcome [49, 54]. Prior to heterogeneity testing, an evaluation of baseline comparability revealed a statistically significant difference in daily sedentary time between the control and intervention groups at baseline. Therefore, a meta-analysis to pool effect sizes was not performed. However, both individual studies reported a statistically significant effect of the intervention in reducing daily sedentary time.

##### Moderate-to-Vigorous Physical Activity (MVPA)

Three studies reported MVPA using varied units [53, 54, 56], with two studies reporting minutes per week [53, 56], one study reporting minutes per day [54]. Compared with control group, intervention had a significant effectiveness on improving MVPA (SMD = 0.40, 95%CI = 0.10, 0.70, *P* < 0.05, Cochran’s Q = 3.89, *p* = 0.14, I^2^ = 49%, fixed model) (Fig. 3C).

#### Physical function

##### Handgrip strength

**Fig. 4:**
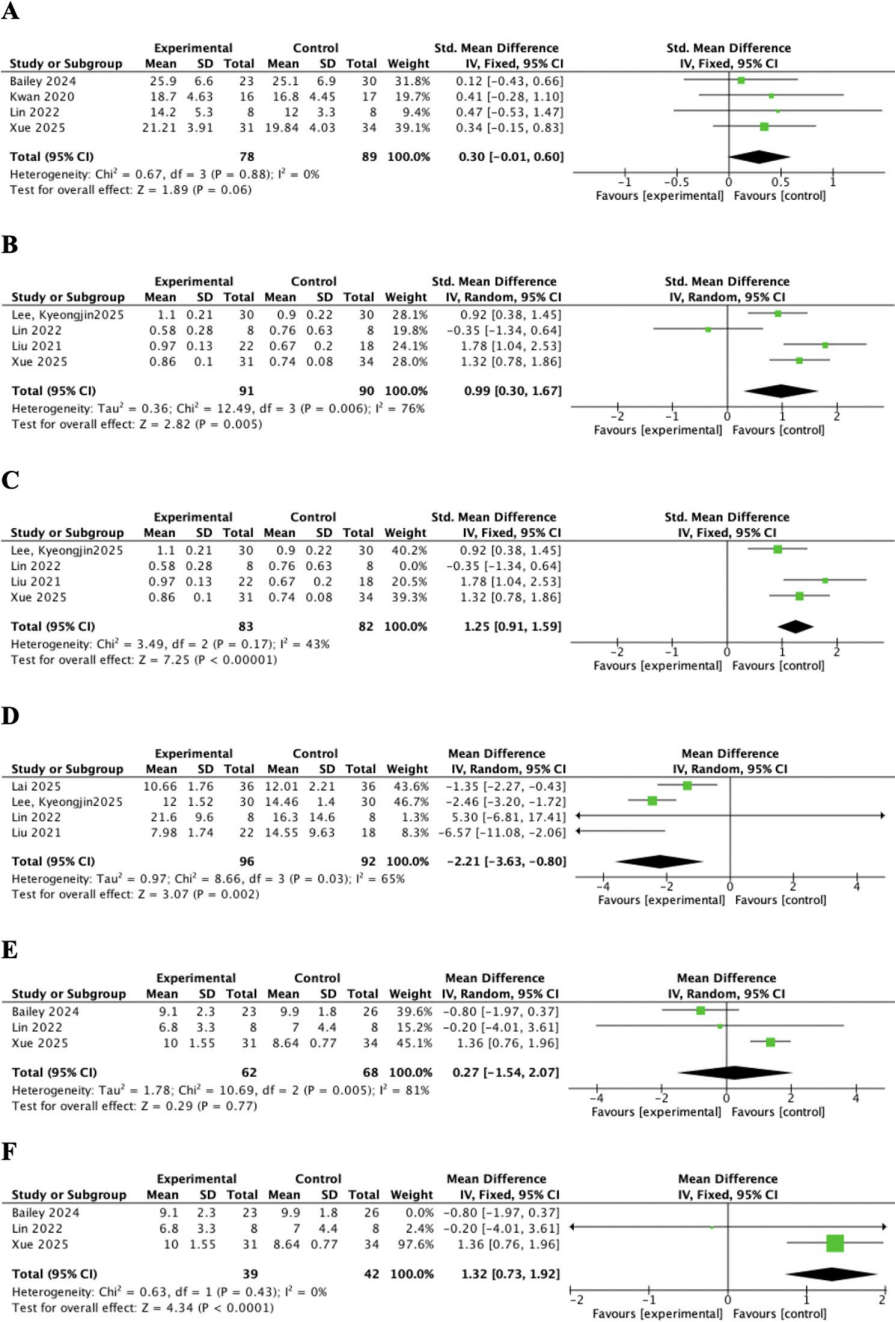
Forest plots for (**A**) hand grip strength; (**B**) gait speed; (**C**) gait speed (sensitivity analysis); (**D**) Timed Up and Go (TUG); (**E**) Short Physical Performance Battery (SPPB); (**F**) SPPB (sensitivity analysis)

Four studies assessed hand grip strength [48, 49, 51, 55]. Compared with the control group, the intervention demonstrated no statistically significant effect on improving handgrip strength (SMD = 0.30, 95%CI = − 0.01, 0.60, *P* = 0.06, Cochran’s Q = 0.67, *p* = 0.88, I² = 0%, fixed model) (Fig. 4A).

##### Gait speed

Four studies assessed gait speed using different methods, but all reported results in meters per second (m/s) [48, 55, 56, 58]. Two studies measured the time to walk 4 m [48, 55], one study recorded the time to walk 10 m [58], and one study assessed the distance covered in a 2-minute walk [56]. Compared with control group, intervention had a significant effectiveness on improving gait speed (SMD = 0.99, 95%CI = 0.30, 1.67, *P* = 0.005, Cochran’s Q = 12.49, *p* = 0.006, I^2^ = 76%, random model) (Fig. 4B). Sensitivity analysis identified the study by Lin, C. C et al. [55] as the source of heterogeneity, leading to a reduction to 43% after its exclusion from analysis. Using a fixed-effect model on the remaining three studies, the pooled analysis also showed a statistically significant effect of the intervention on improving gait speed (SMD = 1.25, 95%CI = 0.91, 1.59, *P* < 0.05, Cochran’s Q = 3.49, *p* = 0.17, I^2^ = 43%) (Fig. 4C).

##### Timed Up and Go (TUG)

Four studies assessed dynamic balance using the TUG [52, 55, 56, 58]. Compared with control group, intervention had a significant effectiveness on improving TUG performance (MD = − 2.21, 95%CI = − 3.63, − 0.80, *P* = 0.002, Cochran’s Q = 8.66, *p* = 0.03, I^2^ = 65%, random model) (Fig. 4D).

##### Short Physical Performance Battery (SPPB)

Three studies assessed physical performance by SPPB [48, 49, 55]. Compared with control group, mHealth intervention had no significant effectiveness on improving SPPB (MD = 0.27, 95%CI = − 1.54, 2.07, *P* = 0.77, Cochran’s Q = 10.69, *p* = 0.005, I^2^ = 81%, random model) (Fig. 4E). Sensitivity analysis identified the study by Bailey, D. P et al. [49] as the source of heterogeneity, leading to a reduction to 0% after its exclusion from analysis. Using a fixed-effect model on the remaining two studies, the pooled analysis showed a statistically significant effect of the intervention on improving SPPB (MD = 1.32, 95%CI = 0.73, 1.92, *P* < 0.05, Cochran’s Q = 0.63, *p* = 0.43, I^2^ = 0%) (Fig. 4F).

#### Exercise self-efficacy

**Fig. 5:**
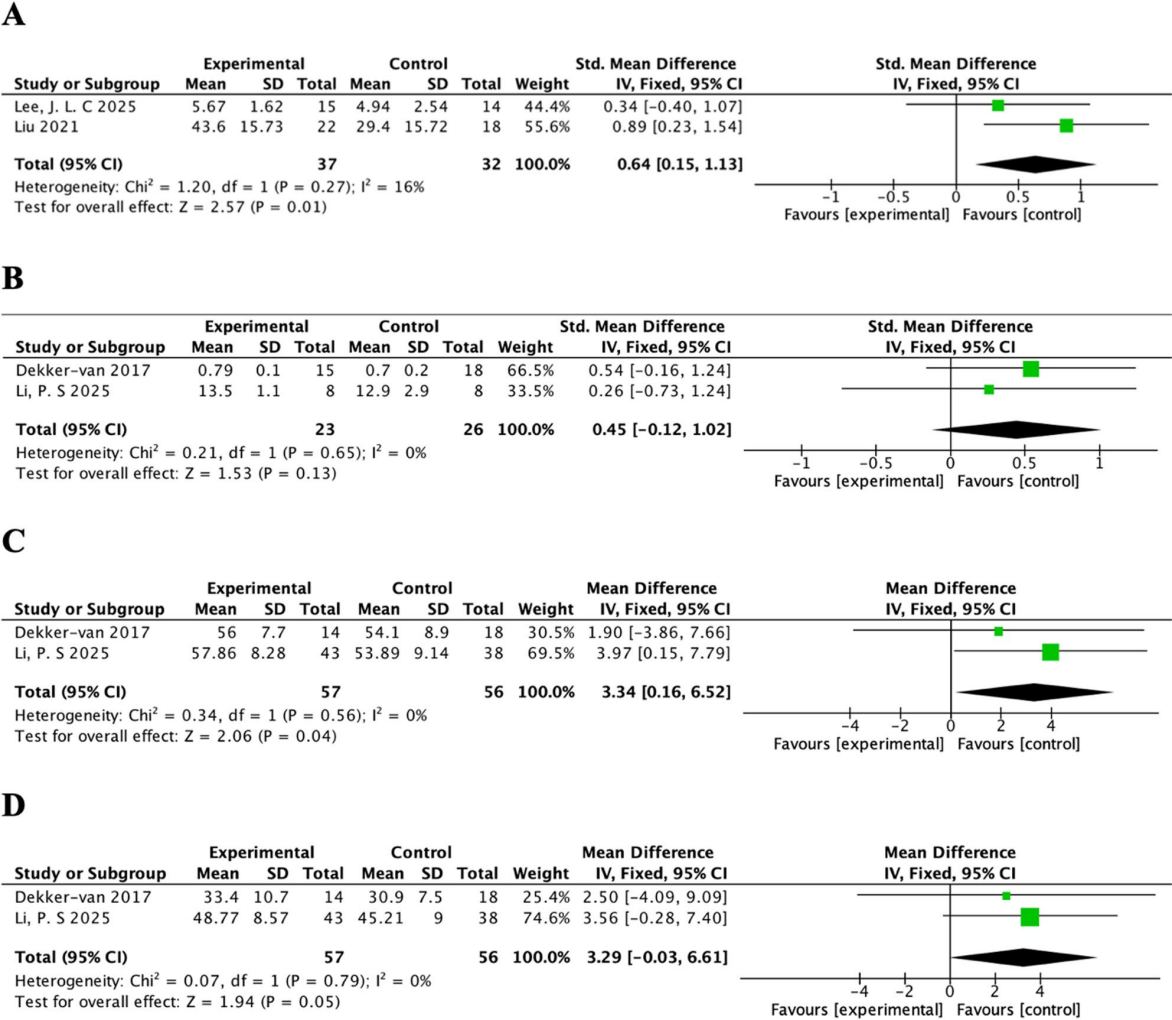
Forest plots for (**A**) exercise self-efficacy; (**B**) global quality of life; (**C**) mental component summary (MCS); (**D**) physical component summary (PCS)

Two studies assessed exercise self-efficacy using the Chinese Version of the Exercise Self-Efficacy Scale (SEE-C) [53, 56], with higher scores indicating greater exercise self-efficacy. Compared with control group, mHealth intervention had a significant effectiveness on improving exercise self-efficacy (SMD = 0.64, 95%CI = 0.15, 1.31, *P* < 0.05, Cochran’s Q = 1.20, *p* = 0.27, I^2^ = 16%, fixed model) (Fig. 5A).

#### Quality of life

Quality of life was evaluated by EuroQol Five-Dimensions Three-Level questionnaire (EQ-5D-3 L) [50, 55], or 12-Item Short-Form Health-Survey (SF-12) [47, 50], which is divided into physical component summary (PCS) and mental component summary (MCS). For global quality of life, the effectiveness of intervention was not significant (SMD = 0.45, 95%CI = − 0.12, 1.02, *P* = 0.13, Cochran’s Q = 0.21, *p* = 65, I^2^ = 0%, fixed model) (Fig. 5B). Regarding domain-specific quality of life, mHealth interventions demonstrated a statistically significant effect on the MCS (MD = 3.34, 95%CI = 0.16, 6.52, *P* = 0.04, Cochran’s Q = 0.34, *p* = 0.56, I^2^ = 0%, fixed model) (Fig. 5C), while no significant effect was observed on the PCS (MD = 3.29, 95%CI = − 0.03, 6.61, *P* = 0.05, Cochran’s Q = 0.07, *p* = 0.79, I^2^ = 0%, fixed model) (Fig. 5D).

### Implementation outcomes

Implementation Outcomes are shown in Supplementary Table 3.

#### Reach

The proportion of eligible participants who were successfully screened and consented to participate was reported in eleven studies, with a median reach rate of 84% (range = 18%–100%).

#### Feasibility

The percentage of participants who were retained for follow-up assessments, with a median score of 93% (range = 74% – 100%).

#### Adherence

A measure of intervention adherence in all studies with a median score of 81%, ranging from 61% to 97%.

#### Safety

According to five studies [47–49, 56, 59], there were no adverse events during the trial (*n* = 5, 35.71% of studies). Among studies documenting incidents, one reported 2 cases (1 fall and 1 case of knee and back pain) [53], while another reported three mild, transient events (knee pain, foot pain, and dizziness) that resolved with rest [54]. A further study noted two incidents (a fall and a fracture) [46]; however, the investigators concluded these were not attributable to the intervention itself. Notably, one trial presented a comparative analysis [57], finding that the intervention group had a significantly lower risk of falls than the control group over a 6-month follow-up (Beta = -0.29, 95% CI: -0.53 to -0.04; *P* = 0.025).

### Publication bias

Funnel plot analysis not performed because the number of studies included for each outcome measure was fewer than ten.

## Discussion

The systematic review and meta-analysis evaluated the effectiveness and implementation of mHealth interventions for older adults with frailty. To our knowledge, this is pioneering research of mHealth interventions targeting individuals for older adults with frailty. The evidence indicates that mHealth interventions can reduce frailty severity when frailty is assessed using continuous measures, increase physical activity levels, and improve selected physical function outcomes, particularly gait speed, TUG, and SPPB. However, the evidence was less consistent for categorical frailty transitions, handgrip strength, and overall quality of life. This pattern suggests that mHealth may first influence behavioural and functional reserve outcomes before producing clear threshold-crossing changes in frailty status.

Active ageing emphasizes maintaining functional ability, independence, and participation in everyday life. In this context, the value of mHealth is not only that it digitizes care, but that it extends physical activity management from clinics into home and community settings through self-monitoring, feedback, reminders, remote coaching, and social support. Our findings therefore suggest that mHealth can serve as a practical vehicle for supporting active and healthy ageing in frail older adults, particularly when the aim is to sustain day-to-day movement and functional maintenance.

### Frailty status

Regarding frailty outcomes, we observed a pattern of significant effects for continuous measures of frailty severity but non-significant effects for categorical frailty transitions. In other words, mHealth interventions appeared to improve frailty scores on validated instruments, but the evidence was insufficient to confirm a statistically significant increase in the probability of moving from one frailty category to a better category within the study periods. When frailty was pooled as a continuous score, mHealth interventions significantly reduced frailty severity. A plausible mechanism is that mHealth via automatic activity tracking through wearables/platforms, tailored reminders and feedback, remote health coaching, and peer support enhances self-monitoring and goal-setting/management, thereby promoting accumulation of daily activity and improving functional reserve [48, 51, 52, 55, 56, 59]. However, when frailty was synthesized as a binary transition outcome (i.e., moving from frailty to pre-frailty/robustness), the difference was not statistically significant. This finding may indicate that within the trial timeframe, mHealth interventions are more likely to yield gradual improvements in frailty-related phenotypes, which are captured by reductions in continuous scores. In contrast, achieving a threshold-crossing change in frailty category may require longer follow-up and more intensive, multidomain interventions [60]. In addition, only two studies contributed to the pooled categorical outcome [47, 57], which may have resulted in limited statistical power and unstable estimates. Notably, in one study enrolling only pre-frail older adults [54], mHealth interventions significantly increased the rate of improvement from pre-frailty to robustness, suggesting greater preventive potential at earlier stages of frailty and underscoring the importance of early identification and timely intervention.

### Physical activity

#### Daily step count

Step count is one of the most commonly used objective indicators of physical activity. After pooling three studies, we found that mHealth interventions showed a near-significant trend toward increasing average daily steps (SMD = 0.57), but the overall effect did not reach statistical significance (*P* = 0.07) and exhibited moderate heterogeneity (I² = 65%). Sensitivity analyses indicated that the study by Bailey et al. [49] was the primary source of heterogeneity. After excluding this study, the pooled effect became statistically significant (SMD = 0.89, *P* < 0.001, I² = 0%). This discrepancy may stem from the limited number of included studies (only three), making the pooled result highly sensitive to individual trials. Notably, Bailey et al.’s trial differed from the other two studies in that its intervention primarily targeted reductions in sedentary behavior (via inactivity alerts) rather than explicitly aiming to increase step counts [49]. This finding underscores the importance of intervention specificity: mHealth tools designed to promote step-count increases (e.g., goal setting and step-based incentives/rewards) are more likely to yield significant improvements [51, 56].

However, the widespread use of step count as a primary outcome also warrants caution. Although step count is intuitive, low-cost, and easily captured by wearable devices, it provides only a partial representation of physical activity in frail older adults. Step count does not reflect activity intensity, movement quality, postural transitions, upper-limb activity, or the context in which movement occurs [61]. In frail populations, a singular focus on numerical step targets may also encourage metric-driven behavior rather than safe and meaningful participation in daily movement. For some individuals, prioritizing higher step counts without adequate consideration of fatigue, balance limitations, pain, or assistive device use may be inappropriate. Therefore, step count should be interpreted as one component of physical activity behavior rather than a standalone indicator of successful intervention.

#### Sedentary behavior

Sedentary behavior is a key modifiable risk factor in frail populations [62]. Importantly, sedentary behavior is not synonymous with physical inactivity. An individual may achieve some planned exercise yet still accumulate prolonged sedentary time across the remainder of the day. This distinction is particularly important in frail older adults, whose daily routines may be characterised not simply by an absence of activity, but by prolonged uninterrupted sedentary time [63]. In the present review, both included studies reported that mHealth interventions significantly reduced daily sedentary time [49, 54]. However, a pooled effect size could not be calculated because there were substantial between-group differences in baseline sedentary time. Nevertheless, the consistent direction of findings suggests that mHealth strategies incorporating real-time feedback and prompts may influence behavioral patterns throughout the day, not merely total activity volume. For example, inactivity alerts, reminders to stand up, and cues to perform brief walking or movement breaks may interrupt prolonged sedentary bouts and redistribute activity across the day. This behavioral-pattern perspective may be especially relevant for frail older adults, for whom reducing prolonged sitting and increasing light-intensity movement may be more achievable and safer initial targets than focusing exclusively on structured exercise or high step-count goals.

#### MVPA

Compared with step count, MVPA more directly reflects an activity dose that promotes cardiorespiratory fitness and functional improvement [64]. Pooling three studies [53, 54, 56], we found that mHealth interventions significantly increased MVPA. Nevertheless, the interpretation of device-measured MVPA in frail older adults requires caution. First, the ecological validity of MVPA estimates may be limited in this population. Many wearable devices and accelerometer cut-points were developed and validated in younger or healthier adults, and may not accurately capture exertion in frail older adults who commonly present with slower gait speed, reduced stride length, altered biomechanics, lower exercise tolerance, and occasional use of walking aids [65]. As a result, activities that are physiologically demanding for a frail older person may not reach conventional device thresholds for MVPA, whereas some detected movements may not necessarily represent purposeful or sustainable health-promoting activity in daily life.

Second, the safety implications of promoting MVPA in frail populations deserve consideration. Although increasing activity intensity may support functional gains, frail older adults often have balance impairment, multimorbidity, pain, fatigue, or elevated fall risk [66, 67]. Therefore, device-detected increases in MVPA should not automatically be interpreted as unequivocally beneficial without considering supervision, progression, symptom response, and adverse events. In this context, the beneficial effects of mHealth may arise not only from increasing intensity, but also from enabling safer and more individualized progression through remote monitoring, tailored feedback, and timely risk alerts.

Third, the definition of MVPA itself may require adaptation in the context of frailty. Standard absolute thresholds may underestimate meaningful exertion in frail older adults, for whom lower absolute workloads may correspond to moderate or even vigorous relative intensity. Future studies should consider complementing device-based MVPA with relative-intensity indicators, perceived exertion, functional capacity, symptom monitoring, and context-specific measures of real-world movement. Such approaches may better reflect clinically meaningful activity in this population than conventional MVPA thresholds alone.

### Physical function

#### Handgrip strength

We did not observe a significant effect of mHealth interventions on handgrip strength. Handgrip strength is an important indicator of overall muscle strength and health status [68, 69], and meaningful improvement typically requires sufficiently intense and targeted resistance-training stimuli [70]. However, most mHealth interventions in the included studies primarily emphasized walking, promotion of daily activities, and low- to moderate-intensity exercise, with limited incorporation of structured resistance-training components [48, 49, 51, 55]. This may be a major reason why improvements in handgrip strength were not statistically significant. Future mHealth programs could incorporate more structured resistance-training modules as core elements (e.g., video-based demonstrations, guidance on the use of resistance tools, and movement feedback) to enhance effects on strength-related outcomes.

#### Gait speed

Gait speed is often regarded as the “sixth vital sign” and has strong predictive value for frailty, disability, and mortality risk [6, 71, 72]. Four studies assessed gait speed using different protocols (with units consistently reported in m/s) [48, 55, 56, 58]. The pooled analysis showed a significant improvement in gait speed with the intervention (SMD = 0.99, 95%CI = 0.30, 1.67, *P* = 0.005, I^2^ = 76%). Sensitivity analyses suggested that the study by Lin et al. [55] was a major source of heterogeneity. After excluding this study, heterogeneity decreased and the effect remained significant (SMD = 1.25, 95%CI = 0.91, 1.59, *P* < 0.05, I^2^ = 43%). The observed improvement may stem from two pathways: first, benefits in physical capacity and gait endurance arising from behavioral changes (increased activity intensity, reduced sedentary time) [73]; second, enhancements in gait control resulting from prescribed interventions (e.g., walking training, balance/gait training videos, remote professional guidance) [74]. The heterogeneity may be attributable to variations in gait speed test protocols (e.g., 6-meter, 10-meter, or 2-minute walk tests) and differences in sample characteristics. The study by Lin et al. [55] had a relatively small sample size (*n* = 16), which may have led to unstable effect estimates and amplified between-study differences. Standardized measurement protocols are recommended for future studies to enhance comparability and minimize study heterogeneity.

#### TUG

The TUG is commonly used to assess mobility and function, especially in older adults. The test assesses balance, gait speed, and mobility by timing the standing up, walking a short distance (usually 3 m or 10 ft), turning, returning to the chair, and sitting down [38]. The American Geriatric Society and the British Geriatric Society [75] recommend the TUG as a routine fall risk screening method. The results showed that mHealth interventions have a significant effect on improving TUG scores. This improvement may be attributable to the fact that most mHealth programs incorporated targeted functional training guidance, such as balance exercise videos and gait correction demonstrations [52].

#### SPPB

Three studies reported the SPPB [48, 49, 55], and the pooled analysis showed no significant improvement (MD = 0.27, 95% CI = − 1.54, 2.07, *P* = 0.77; I² = 81%). However, sensitivity analyses indicated that after excluding the study by Bailey et al. [49], heterogeneity decreased to 0% and the intervention effect became significant (MD = 1.32, 95% CI = 0.73, 1.92, *P* < 0.05). This discrepancy may be explained by the small number of included studies (*n* = 3), which makes the pooled estimate highly sensitive to individual trials. Bailey et al.’s [49] intervention primarily targeted sedentary behavior rather than comprehensive physical function training. Because the SPPB captures three domains, namely balance, gait speed, and lower-extremity strength [75], meaningful improvements typically require domain-specific training, and an intervention focused on a single domain is unlikely to meaningfully improve composite measures such as the SPPB.

### Exercise self-efficacy

The meta-analysis results suggest that mHealth interventions significantly improve exercise self-efficacy. For frail older adults, improved self-efficacy translates into greater confidence to act, stronger self-management capacity, and a more stable intention to persist [76], which are precisely the aspects that are most difficult to sustain through traditional short-term, face-to-face exercise guidance. By providing ongoing feedback, staged goals, visualized progress, and timely encouragement, mHealth interventions may help progressively shift reliance on external supervision toward internal motivation, thereby enhancing the sustainability of long-term management [77]. These findings also offer a plausible explanation for the behavior–function–frailty pathway: gains in self-efficacy may be an important mediating pathway linking increased physical activity to functional improvement. Future research is recommended to employ mechanism-testing approaches, such as mediation analysis or structural equation modeling, to further clarify its role.

### Quality of life

mHealth interventions did not demonstrate a significant improvement in overall quality of life. However, at the domain level, the MCS improved significantly, whereas the PCS showed no significant difference. The significant improvement in MCS may be attributable to two factors. First, regular physical activity can modulate neurotransmitters, such as dopamine and serotonin, thereby alleviating depressive and anxiety symptoms [78]. Second, social isolation is a major contributor to declining mental health in older adults [79], and mHealth technologies may mitigate social isolation, particularly among community-dwelling and long-term care populations, by facilitating emotional support through video calls and group-based communication [80].

In contrast, the lack of significant improvement in PCS may have several explanations. One possible reason is the relatively short duration of most included interventions, the majority of which lasted 24 weeks or less. In frail older adults, improvements in the physical component of quality of life may take longer to emerge than improvements in mental well-being, because this domain often depends on sustained gains in mobility, endurance, symptom relief, and independence in daily functioning [81]. In addition, meaningful improvement in this physical dimension may require multidomain interventions [82], such as nutritional support and comorbidity management [83], whereas most interventions in this review focused solely on physical activity without integrating additional supportive components.

### Implementation of mHealth interventions

In the health management of older adults with frailty, utilizing mHealth interventions to promote physical activities may become an effective and acceptable way of promoting health. The included studies in this systematic review generally demonstrated good feasibility, with median reach and feasibility rates of 84% (range: 18% – 100%) and 93% (range: 74% – 100%), respectively. However, the wide variation in reach, with rates as low as 18%, suggests that uptake of mHealth interventions among frail older adults may be influenced by multiple factors. These may include differences in digital literacy, access to smartphones or internet-enabled devices, recruitment settings, intervention complexity, and the availability of caregiver or family support. Such findings suggest that future mHealth programs should prioritize user-centred design, simplified interfaces, stratified training, caregiver/family involvement, and early user needs assessments to enhance accessibility and uptake.

It is noteworthy that there is currently little consensus on how to define and measure adherence to mHealth interventions [31]. A recent systematic review on mHealth interventions for promoting adherence and self-management in chronic disease patients highlighted the lack of agreement on the optimal level of user engagement required to facilitate meaningful behavioral change. The review emphasized the need to establish minimum engagement research benchmarks to scientifically represent the level at which participants are sufficiently, acceptably, or meaningfully engaged in the intervention [32]. The adherence assessment criteria varied across studies included in this review, yet the findings consistently demonstrated satisfactory adherence to mHealth interventions (89%, range from 74% to 100%). Future research should establish more explicit adherence evaluation standards to identify effective adherence-enhancing strategies and facilitate broader implementation of such interventions.

Moreover, we are unable to remark on any negative effects connected to mHealth interventions because only a limited number of studies have reported adverse events, including exercise-induced pain, or falls. These findings suggested that while mHealth interventions appear to be generally safe for older adults with frailty, personal situations, such as pre-existing physical conditions, must be carefully considered when designing and implementing such procedures. Future research should prioritize addressing these gaps through monitoring and reporting of adverse events throughout intervention implementation. Researchers could also conduct subgroup analyses based on participants’ pre-existing health conditions to identify risk-stratified outcomes, while implementing long-term follow-up protocols to detect delayed adverse reactions that may not become evident during short-term assessments. These comprehensive approaches will strengthen our understanding of safety for mHealth interventions, ultimately improving the promotion of the intervention.

Taken together, the findings on feasibility, adherence, and safety also have important practical implications for clinicians and healthcare systems considering the implementation of mHealth interventions for frail older adults. Implementation should be individualized according to frailty severity, functional status, comorbidities, safety risk, and digital literacy, rather than delivered as a uniform approach. Prior to initiation, assessment of patients’ access to devices or internet connection, ability to use digital tools, and availability of caregiver support may help identify potential barriers and guide the level of assistance required. During implementation, combining simple and accessible platforms with structured onboarding, regular feedback, and timely professional follow-up may enhance engagement and long-term adherence.

### Limitations and potential directions for future research

This systematic review and meta-analysis has several limitations. First, this review included studies published in English and Chinese only. Although no eligible studies published in other languages were identified during the search and screening process, relevant studies in other languages may still have been missed because of database coverage and language-related indexing limitations, which may have introduced language bias and reduced the comprehensiveness of the evidence base. In addition, selection bias may have existed at the primary-study level, as older adults who were willing to participate in mHealth interventions may have differed systematically from those who declined, particularly in terms of digital literacy, access to devices or internet services, and motivation to engage in health management. This may have led to overestimation of the feasibility, adherence, and effectiveness of mHealth interventions and may limit the generalizability of our findings to frail older adults with lower digital readiness. Second, most included studies (92%) were judged to be at high risk of bias with respect to blinding of participants and personnel. Inadequate blinding may have exaggerated the effects on subjective or behavior-related outcomes. Third, several meta-analyses were based on only a small number of studies. When the number of included studies is small, the summary effect may be unstable, confidence intervals may be imprecise, and the results can be disproportionately influenced by a single study. Moreover, because the number of studies pooled for each outcome was generally fewer than ten, formal assessment of publication bias using methods such as funnel plots was not possible. Therefore, these findings should be regarded as exploratory rather than conclusive, and more adequately powered, methodologically rigorous trials are needed before firm inferences can be made in the future. Fourth, due to the limited number of studies, we were unable to perform subgroup analyses based on baseline frailty severity (i.e., frail vs. pre-frail), which may have concealed differential intervention effects. Future studies with larger sample sizes should investigate whether intervention effectiveness varies by frailty stage, enabling more tailored mHealth approaches. Fifth, the included studies were relatively concentrated in China. Differences across countries or regions in digital literacy, technology accessibility, and care systems may influence the scalability and effectiveness of mHealth interventions. Future research should consider conducting multicenter, cross-cultural validation studies and optimizing accessibility to bridge the digital divide. Sixth, evidence on long-term outcomes and cost-effectiveness remains insufficient. The public health value of frailty interventions extends beyond short-term improvements in physical performance to longer-term reductions in falls, hospitalizations, disability, and caregiving burden. Future studies should incorporate longer follow-up periods and include health economic evaluations.

## Conclusion

This systematic review and meta-analysis suggests that mHealth interventions have the potential to increase physical activity levels, reduce frailty severity as measured by continuous indices, and improve selected physical function outcomes (gait speed, TUG, and SPPB) among frail older adults, with promising feasibility and safety profiles. However, evidence regarding categorical frailty transitions, handgrip strength, and overall quality of life remains limited and should be further validated and strengthened by high-quality, standardized RCTs with longer follow-up. Given the limited number of included studies, caution is warranted when interpreting the findings of this investigation. Future research should prioritize studies with rigorous methodologies and larger sample sizes to further strengthen the evidence base for mHealth interventions on physical activity management targeting older adults with frailty.

## Supplementary Information

Supplementary Material 1.

## Data Availability

No datasets were generated or analysed during the current study.

## References

[1] Fried LP, Ferrucci L, Darer J, Williamson JD, Anderson G. Untangling the concepts of disability, frailty, and comorbidity: implications for improved targeting and care. J Gerontol Biol Sci Med Sci. 2004;59(3):255–63. 10.1093/gerona/59.3.m255. 10.1093/gerona/59.3.m255 15031310

[2] O’Caoimh R, Sezgin D, O’Donovan MR, Molloy DW, Clegg A, Rockwood K, et al. Prevalence of frailty in 62 countries across the world: a systematic review and meta-analysis of population-level studies. Age Ageing. 2021;50(1):96–104. 10.1093/ageing/afaa219. 33068107 10.1093/ageing/afaa219

[3] Yamaguchi R, Makino K, Katayama O, Yamagiwa D, Shimada H. Relationship between self-rated health, physical frailty, and incidence of disability among Japanese community-dwelling older adults: A longitudinal prospective cohort study. Prev Med. 2025;191:108210. 10.1016/j.ypmed.2024.108210. 39694103 10.1016/j.ypmed.2024.108210

[4] Matsue Y, Kamiya K, Saito H, Saito K, Ogasahara Y, Maekawa E, et al. Prevalence and prognostic impact of the coexistence of multiple frailty domains in elderly patients with heart failure: the FRAGILE-HF cohort study. Eur J Heart Fail. 2020;22(11):2112–9. 10.1002/ejhf.1926. 32500539 10.1002/ejhf.1926

[5] Bray NW, Smart RR, Jakobi JM, Jones GR. Exercise prescription to reverse frailty. Appl Physiol Nutr Metab. 2016;41(10):1112–6. 10.1139/apnm-2016-0226. 27649859 10.1139/apnm-2016-0226

[6] Fried LP, Tangen CM, Walston J, Newman AB, Hirsch C, Gottdiener J, et al. Frailty in older adults: evidence for a phenotype. J Gerontol Biol Sci Med Sci. 2001;56(3):M146–56. 10.1093/gerona/56.3.m146. 10.1093/gerona/56.3.m146 11253156

[7] Rockwood K, Song X, MacKnight C, Bergman H, Hogan DB, McDowell I, et al. A global clinical measure of fitness and frailty in elderly people. CMAJ. 2005;173(5):489–95. 10.1503/cmaj.050051. 16129869 10.1503/cmaj.050051 PMC1188185

[8] Clegg A, Young J, Iliffe S, Rikkert MO, Rockwood K. Frailty in elderly people. Lancet. 2013;381(9868):752–62. 10.1016/s0140-6736(12)62167-9. 23395245 10.1016/S0140-6736(12)62167-9 PMC4098658

[9] Gobbens RJ, van Assen MA, Luijkx KG, Wijnen-Sponselee MT, Schols JM. The Tilburg Frailty Indicator: psychometric properties. J Am Med Dir Assoc. 2010;11(5):344–55. 10.1016/j.jamda.2009.11.003. 20511102 10.1016/j.jamda.2009.11.003

[10] Zhao W, Hu P, Sun W, Wu W, Zhang J, Deng H, et al. Effect of physical activity on the risk of frailty: A systematic review and meta-analysis. PLoS ONE. 2022;17(12):e0278226. 10.1371/journal.pone.0278226. 36454790 10.1371/journal.pone.0278226 PMC9714708

[11] Dent E, Morley JE, Cruz-Jentoft AJ, Woodhouse L, Rodríguez-Mañas L, Fried LP, et al. Physical Frailty: ICFSR International Clinical Practice Guidelines for Identification and Management. J Nutr Health Aging. 2019;23(9):771–87. 10.1007/s12603-019-1273-z. 31641726 10.1007/s12603-019-1273-z PMC6800406

[12] LiQ, Guan J, Wang R. Association of physical activity levels with frailty index in elderly Chinese: evidence from the China health and retirement longitudinal study (CHARLS). 1471–2318 (Electronic). 10.1186/s12877-025-06334-5. 10.1186/s12877-025-06334-5 PMC12506303 41057757

[13] Dent E, Lien C, Lim WS, Wong WC, Wong CH, Ng TP, et al. The Asia-Pacific Clinical Practice Guidelines for the Management of Frailty. J Am Med Dir Assoc. 2017;18(7):564–75. 10.1016/j.jamda.2017.04.018. 28648901 10.1016/j.jamda.2017.04.018

[14] Caspersen CJ, Powell KE, Christenson GM. Physical activity, exercise, and physical fitness: definitions and distinctions for health-related research. Public Health Rep. 1985;100(2):126–31. 3920711 PMC1424733

[15] Bull FC, Al-Ansari SS, Biddle S, Borodulin K, Buman MP, Cardon G, et al. World Health Organization 2020 guidelines on physical activity and sedentary behaviour. Br J Sports Med. 2020;54(24):1451–62. 10.1136/bjsports-2020-102955. 33239350 10.1136/bjsports-2020-102955 PMC7719906

[16] Li Q, Guan J, Wang R. Association of physical activity levels with frailty index in elderly Chinese: evidence from the China health and retirement longitudinal study (CHARLS). BMC Geriatr. 2025;25(1):762. 10.1186/s12877-025-06334-5. 41057757 10.1186/s12877-025-06334-5 PMC12506303

[17] Tremblay MS, Aubert S, Barnes JD, Saunders TJ, Carson V, Latimer-Cheung AE, et al. Sedentary Behavior Research Network (SBRN) - Terminology Consensus Project process and outcome. Int J Behav Nutr Phys Act. 2017;14(1):75. 10.1186/s12966-017-0525-8. 28599680 10.1186/s12966-017-0525-8 PMC5466781

[18] World Health Organization. WHO guidelines on physical activity and sedentary behaviour. Geneva: World Health Organization; 2020. ISBN: 9789240015128.

[19] Franco MR, Tong A, Howard K, Sherrington C, Ferreira PH, Pinto RZ, et al. Older people’s perspectives on participation in physical activity: a systematic review and thematic synthesis of qualitative literature. Br J Sports Med. 2015;49(19):1268–76. 10.1136/bjsports-2014-094015. 25586911 10.1136/bjsports-2014-094015

[20] Meredith SJ, Cox NJ, Ibrahim K, Higson J, McNiff J, Mitchell S, et al. Factors that influence older adults’ participation in physical activity: a systematic review of qualitative studies. Age Ageing. 2023;52(8). 10.1093/ageing/afad145. 10.1093/ageing/afad145 PMC10438214 37595070

[21] Henderson RM, Miller ME, Fielding RA, Gill TM, Glynn NW, Guralnik JM, et al. Maintenance of Physical Function 1 Year After Exercise Intervention in At-Risk Older Adults: Follow-up From the LIFE Study. J Gerontol Biol Sci Med Sci. 2018;73(5):688–94. 10.1093/gerona/glx231. 10.1093/gerona/glx231 PMC5905630 29490012

[22] Yerrakalva D, Yerrakalva D, Hajna S, Griffin S. Effects of Mobile Health App Interventions on Sedentary Time, Physical Activity, and Fitness in Older Adults: Systematic Review and Meta-Analysis. J Med Internet Res. 2019;21(11):e14343. 10.2196/14343. 31778121 10.2196/14343 PMC6908977

[23] Varshney U. Mobile health: Four emerging themes of research. Decis Support Syst. 2014;66:20–35. 10.1016/j.dss.2014.06.001.

[24] Zhang R, Wang H. Insights into the technological evolution and research trends of mobile health: bibliometric analysis. LID – 10.3390/healthcare13070740 [doi] LID – 740. 2227–9032 (Print). 10.3390/healthcare13070740. 10.3390/healthcare13070740 PMC11988424 40218038

[25] Triantafyllidis A, Kondylakis H, Votis K, Tzovaras D, Maglaveras N, Rahimi K. Features, outcomes, and challenges in mobile health interventions for patients living with chronic diseases: a review of systematic reviews. 1872–8243 (Electronic). 10.1016/j.ijmedinf.2019.103984. 10.1016/j.ijmedinf.2019.103984 31605884

[26] Sohaib Aslam A, van Luenen S, Aslam S, van Bodegom D, Chavannes NH. A systematic review on the use of mHealth to increase physical activity in older people. Clin eHealth. 2020;3:31–9. 10.1016/j.ceh.2020.04.002.

[27] Tabira KA-O, Oguma YA-O, Yoshihara SA-O, Shibuya MA-O, Nakamura MA-O, Doihara NA-O, et al. Digital peer-supported app intervention to promote physical activity among community-dwelling older adults: nonrandomized controlled trial. 2561–7605 (Electronic). 10.2196/56184. 10.2196/56184 PMC11176879 38814686

[28] Esfandiari E, Miller WC, Ashe MC. The Effect of Telehealth Interventions on Function and Quality of Life for Older Adults with Pre-Frailty or Frailty: A Systematic Review and Meta-Analysis. J Appl Gerontol. 2021;40(11):1649–58. 10.1177/0733464820983630. 33402043 10.1177/0733464820983630

[29] Dawson R, Oliveira JS, Kwok WS, Bratland M, Rajendran IM, Srinivasan A, et al. Exercise Interventions Delivered Through Telehealth to Improve Physical Functioning for Older Adults with Frailty, Cognitive, or Mobility Disability: A Systematic Review and Meta-Analysis. Telemed J E Health. 2024;30(4):940–50. 10.1089/tmj.2023.0177. 37975811 10.1089/tmj.2023.0177 PMC11035924

[30] Han HW, Park SW, Kim DY, Lee BS, Kim D, Jeon N, et al. E-Health Interventions for Older Adults With Frailty: A Systematic Review. Ann Rehabil Med. 2023;47(5):348–57. 10.5535/arm.23090. 37907226 10.5535/arm.23090 PMC10620492

[31] Yang Y, Boulton E, Todd C. Measurement of Adherence to mHealth Physical Activity Interventions and Exploration of the Factors That Affect the Adherence: Scoping Review and Proposed Framework. J Med Internet Res. 2022;24(6):e30817. 10.2196/30817. 35675111 10.2196/30817 PMC9218881

[32] Eaton C, Vallejo N, McDonald X, Wu J, Rodríguez R, Muthusamy N, et al. User Engagement With mHealth Interventions to Promote Treatment Adherence and Self-Management in People With Chronic Health Conditions: Systematic Review. J Med Internet Res. 2024;26:e50508. 10.2196/50508. 39316431 10.2196/50508 PMC11462107

[33] Page MJ, McKenzie JE, Bossuyt PM, Boutron I, Hoffmann TC, Mulrow CD, et al. The PRISMA 2020 statement: an updated guideline for reporting systematic reviews. BMJ. 2021;372:n71. 10.1136/bmj.n71. 33782057 10.1136/bmj.n71 PMC8005924

[34] Ensrud KE, Ewing SK, Taylor BC, Fink HA, Cawthon PM, Stone KL, et al. Comparison of 2 Frailty Indexes for Prediction of Falls, Disability, Fractures, and Death in Older Women. Arch Intern Med. 2008;168(4):382–9. 10.1001/archinternmed.2007.113. 18299493 10.1001/archinternmed.2007.113

[35] Newman AB, Gottdiener JS, McBurnie MA, Hirsch CH, Kop WJ, Tracy R, et al. Associations of subclinical cardiovascular disease with frailty. J Gerontol Biol Sci Med Sci. 2001;56(3):M158–66. 10.1093/gerona/56.3.m158. 10.1093/gerona/56.3.m158 11253157

[36] Morley JE, Malmstrom TK, Miller DK. A simple frailty questionnaire (FRAIL) predicts outcomes in middle aged African Americans. J Nutr Health Aging. 2012;16(7):601–8. 10.1007/s12603-012-0084-2. 22836700 10.1007/s12603-012-0084-2 PMC4515112

[37] Peters LL, Boter H, Buskens E, Slaets JP. Measurement properties of the Groningen Frailty Indicator in home-dwelling and institutionalized elderly people. J Am Med Dir Assoc. 2012;13(6):546–51. 10.1016/j.jamda.2012.04.007. 22579590 10.1016/j.jamda.2012.04.007

[38] Podsiadlo D, Richardson S. The timed Up & Go: a test of basic functional mobility for frail elderly persons. J Am Geriatr Soc. 1991;39(2):142–8. 10.1111/j.1532-5415.1991.tb01616.x. 1991946 10.1111/j.1532-5415.1991.tb01616.x

[39] Guralnik JM, Simonsick EM, Ferrucci L, Glynn RJ, Berkman LF, Blazer DG, et al. A short physical performance battery assessing lower extremity function: association with self-reported disability and prediction of mortality and nursing home admission. J Gerontol. 1994;49(2):M85–94. 10.1093/geronj/49.2.m85. 8126356 10.1093/geronj/49.2.m85

[40] Badia X, Roset M, Montserrat S, Herdman M, Segura A. [The Spanish version of EuroQol: a description and its applications. Eur Qual Life scale] Med Clin (Barc). 1999;112(Suppl 1):79–85. 10618804

[41] Ware J Jr., Kosinski M, Keller SD. A 12-Item Short-Form Health Survey: construction of scales and preliminary tests of reliability and validity. Med Care. 1996;34(3):220–33. 10.1097/00005650-199603000-00003. 8628042 10.1097/00005650-199603000-00003

[42] Cumpston M, Li T, Page MJ, Chandler J, Welch VA, Higgins JP, et al. Updated guidance for trusted systematic reviews: a new edition of the Cochrane Handbook for Systematic Reviews of Interventions. Cochrane Database Syst Rev. 2019;10(10):Ed000142. 10.1002/14651858.Ed000142. 31643080 10.1002/14651858.ED000142 PMC10284251

[43] Tufanaru C, Munn Z, Aromataris E, Campbell J, Hopp L. Chapter 3: Systematic reviews of effectiveness. In: Aromataris E, Munn Z, editors. JBI manual for evidence synthesis. Adelaide: Joanna Briggs Institute; 2019.

[44] Higgins JP, Thompson SG, Deeks JJ, Altman DG. Measuring inconsistency in meta-analyses. BMJ. 2003;327(7414):557–60. 10.1136/bmj.327.7414.557. 12958120 10.1136/bmj.327.7414.557 PMC192859

[45] DeeksJJ, Higgins JP, Altman DG, Group CSM. Analysing data and undertaking meta-analyses. Cochrane handbook for systematic reviews of interventions. 2019:241–84. 10.1002/9781119536604.ch10.

[46] Piau A, Steinmeyer Z, Charlon Y, Courbet L, Rialle V, Lepage B, et al. A Smart Shoe Insole to Monitor Frail Older Adults’ Walking Speed: Results of Two Evaluation Phases Completed in a Living Lab and Through a 12-Week Pilot Study. JMIR Mhealth Uhealth. 2021;9(7):e15641. 10.2196/15641. 36260404 10.2196/15641 PMC8406107

[47] Li PS, Hsieh CJ, Miao NF, Tsai CH, Liu CY, Lin HR, et al. Enhancing Frailty Status and Health-Related Quality of Life in Community-Dwelling Frail Older Adults. Gerontology. 2025;71(4):273–91. 10.1159/000543909. 40552877 10.1159/000543909

[48] Xue J, Zhou Y, Yan Y, Mao Q, Lin F, Shen L, et al. Effects of nurse-led cognitive-motor dual-task training based on mobile health technology on the older adults with cognitive frailty: a quasi-experimental study. Volume 61. New York, NY: Geriatric nursing; 2025. pp. 544–53. 10.1016/j.gerinurse.2024.12.013. 10.1016/j.gerinurse.2024.12.013 39742543

[49] Bailey DP, Harper JH, Kilbride C, McGowan LJ, Victor C, Brierley ML, et al. The frail-LESS (LEss sitting and sarcopenia in frail older adults) remote intervention to improve sarcopenia and maintain independent living via reductions in sedentary behaviour: findings from a randomised controlled feasibility trial. BMC Geriatr. 2024;24(1):747. 10.1186/s12877-024-05310-9. 39251904 10.1186/s12877-024-05310-9 PMC11382500

[50] Dekker-van Weering M, Jansen-Kosterink S, Frazer S, Vollenbroek-Hutten M, User, Experience. Actual Use, and Effectiveness of an Information Communication Technology-Supported Home Exercise Program for Pre-Frail Older Adults. Front Med (Lausanne). 2017;4:208. 10.3389/fmed.2017.00208. 29250523 10.3389/fmed.2017.00208 PMC5715376

[51] Kwan RY, Lee D, Lee PH, Tse M, Cheung DS, Thiamwong L, et al. Effects of an mHealth Brisk Walking Intervention on Increasing Physical Activity in Older People With Cognitive Frailty: Pilot Randomized Controlled Trial. JMIR Mhealth Uhealth. 2020;8(7):e16596. 10.2196/16596. 32735218 10.2196/16596 PMC7428907

[52] Lai X, Zhu H, Cai Y, Chen B, Li Y, Du H, et al. Effects of exercise-cognitive dual-task training on cognitive frailty in older adults: a randomized controlled trial. Front Aging Neurosci. 2025;17:1639245. 10.3389/fnagi.2025.1639245. 41035820 10.3389/fnagi.2025.1639245 PMC12479488

[53] Lee JLC, Wong AYL, Ng PHF, Fu SN, Fong KNK, Cheng ASK, et al. Outdoor Exercise Facility-Based Integrative Mobile Health Intervention to Support Physical Activity, Mental Well-Being, and Exercise Self-Efficacy Among Older Adults With Prefrailty and Frailty in Hong Kong: Pilot Feasibility Randomized Controlled Trial Study. JMIR Mhealth Uhealth. 2025;13:e69259. 10.2196/69259. 40471669 10.2196/69259 PMC12179572

[54] Li N, Wang N, Xu Y, Lin S, Yuan Y, Huang F, et al. The impacts of a mHealth platform-enabled lifestyle-integrated multicomponent exercise program on reversing pre-frailty in community-dwelling older adults: A randomized controlled trial. Int J Nurs Stud. 2025;167:105072. 10.1016/j.ijnurstu.2025.105072. 40222237 10.1016/j.ijnurstu.2025.105072

[55] Lin CC, Kuo LC, Lin YS, Chang CM, Hu FW, Chen YJ, et al. AIoT-Based Ergometer for Physical Training in Frail Elderly with Cognitive Decline: A Pilot Randomized Control Trial. J Med Biol Eng. 2022;42(6):909–21. 10.1007/s40846-022-00759-8.

[56] Liu JYW, Kwan RYC, Yin YH, Lee PH, Siu JY, Bai X. Enhancing the Physical Activity Levels of Frail Older Adults with a Wearable Activity Tracker-Based Exercise Intervention: A Pilot Cluster Randomized Controlled Trial. Int J Environ Res Public Health. 2021;18:19. 10.3390/ijerph181910344. 10.3390/ijerph181910344 PMC8507976 34639644

[57] Valdés-Aragonés M, Pérez-Rodríguez R, Carnicero JA, Moreno-Sánchez PA, Oviedo-Briones M, Villalba-Mora E, et al. Effects of monitoring frailty through a mobile/web-based application and a sensor kit to prevent functional decline in frail and prefrail older adults: FACET (frailty care and well function) pilot randomized controlled trial. J Med Internet Res. 2024;26:e58312. 39436684 10.2196/58312 PMC11538877

[58] Lee K. Effects of remote exercise on physical function in pre-frail older adults: a randomized controlled trial. Med Sci Monitor: Int Med J Experimental Clin Res. 2025;31:e947105. 10.12659/MSM.947105 PMC11786508 39871464

[59] Zhang J, Shi H, Li J, Shen J, Liu X, Zhao F. The effect of a multi-component exercise training program on improving the severity of frailty and physical function of the elderly. Chin J Geriatr. 2023:1201–6. 10.3760/cma.j.issn.0254-9026.2023.10.010.

[60] Dedeyne L, Deschodt M, Verschueren S, Tournoy J, Gielen E. Effects of multi-domain interventions in (pre)frail elderly on frailty, functional, and cognitive status: a systematic review. Clin Interv Aging. 2017;12:873–96. 10.2147/cia.S130794. 28579766 10.2147/CIA.S130794 PMC5448695

[61] Latorre-Román PÁ, de la Casa-Pérez A, Párraga-Montilla JA, Salas-Sánchez J, Lucena-Zurita M, Cabrera-Linares JC. Limitations of Daily Step Count for Assessing Health in Older Adults: The Need to Consider Walking Intensity. Epidemiologia. 2026;7(1):24. 41718056 10.3390/epidemiologia7010024 PMC12922111

[62] Battista F, Duregon F, Vecchiato M, Ermolao A, Neunhaeuserer D. Sedentary lifestyle and physical inactivity: A mutual interplay with early and overt frailty. Nutr Metab Cardiovasc Dis. 2025;35(6):103971. 10.1016/j.numecd.2025.103971. 40180827 10.1016/j.numecd.2025.103971

[63] Kikuchi H, Inoue S, Amagasa S, Fukushima N, Machida M, Murayama H, et al. Associations of older adults’ physical activity and bout-specific sedentary time with frailty status: Compositional analyses from the NEIGE study. Exp Gerontol. 2021;143:111149. 10.1016/j.exger.2020.111149. 33181316 10.1016/j.exger.2020.111149

[64] Lai TF, Liao Y, Lin CY, Huang WC, Hsueh MC, Chan DC. Moderate-to-vigorous physical activity duration is more important than timing for physical function in older adults. Sci Rep. 2020;10(1):21344. 10.1038/s41598-020-78072-0. 33288797 10.1038/s41598-020-78072-0 PMC7721720

[65] Zhang X, Li F, Hobbelen HS, van Munster BC, Lamoth CJ. Gait parameters and daily physical activity for distinguishing pre-frail, frail, and non-frail older adults: A scoping review. J Nutr Health Aging. 2025;29(7):100580. 10.1016/j.jnha.2025.100580. 40373391 10.1016/j.jnha.2025.100580 PMC12173000

[66] Vetrano DL, Palmer K, Marengoni A, Marzetti E, Lattanzio F, Roller-Wirnsberger R, et al. Frailty and Multimorbidity: A Systematic Review and Meta-analysis. J Gerontol Biol Sci Med Sci. 2019;74(5):659–66. 10.1093/gerona/gly110. 10.1093/gerona/gly110 29726918

[67] Yang ZC, Lin H, Jiang GH, Chu YH, Gao JH, Tong ZJ, et al. Frailty Is a Risk Factor for Falls in the Older Adults: A Systematic Review and Meta-Analysis. J Nutr Health Aging. 2023;27(6):487–595. 10.1007/s12603-023-1935-8. 37357334 10.1007/s12603-023-1935-8 PMC12880030

[68] Cruz-Jentoft AJ, Bahat G, Bauer J, Boirie Y, Bruyère O, Cederholm T, et al. Sarcopenia: revised European consensus on definition and diagnosis. Age Ageing. 2019;48(1):16–31. 10.1093/ageing/afy169. 30312372 10.1093/ageing/afy169 PMC6322506

[69] Soysal P, Hurst C, Demurtas J, Firth J, Howden R, Yang L, et al. Handgrip strength and health outcomes: Umbrella review of systematic reviews with meta-analyses of observational studies. J Sport Health Sci. 2021;10(3):290–5. 10.1016/j.jshs.2020.06.009. 32565244 10.1016/j.jshs.2020.06.009 PMC8167328

[70] Grgic J, Garofolini A, Orazem J, Sabol F, Schoenfeld BJ, Pedisic Z. Effects of Resistance Training on Muscle Size and Strength in Very Elderly Adults: A Systematic Review and Meta-Analysis of Randomized Controlled Trials. Sports Med. 2020;50(11):1983–99. 10.1007/s40279-020-01331-7. 32740889 10.1007/s40279-020-01331-7

[71] Fritz S, Lusardi M. White paper:walking speed: the sixth vital sign. J Geriatr Phys Ther. 2009;32(2):2–5. 20039582

[72] Perera S, Patel KV, Rosano C, Rubin SM, Satterfield S, Harris T, et al. Gait Speed Predicts Incident Disability: A Pooled Analysis. J Gerontol Biol Sci Med Sci. 2016;71(1):63–71. 10.1093/gerona/glv126. 10.1093/gerona/glv126 PMC4715231 26297942

[73] Fielding RA, Guralnik JM, King AC, Pahor M, McDermott MM, Tudor-Locke C, et al. Dose of physical activity, physical functioning and disability risk in mobility-limited older adults: Results from the LIFE study randomized trial. PLoS ONE. 2017;12(8):e0182155. 10.1371/journal.pone.0182155. 28820909 10.1371/journal.pone.0182155 PMC5562326

[74] He Z, Wu H, Zhao G, Zhang Y, Li C, Xing Y, et al. The effectiveness of digital technology-based Otago Exercise Program on balance ability, muscle strength and fall efficacy in the elderly: a systematic review and meta-analysis. BMC Public Health. 2025;25(1):71. 10.1186/s12889-024-21251-9. 39773392 10.1186/s12889-024-21251-9 PMC11707927

[75] Summary of the Updated American Geriatrics Society/British Geriatrics. Society clinical practice guideline for prevention of falls in older persons. J Am Geriatr Soc. 2011;59(1):148–57. 10.1111/j.1532-5415.2010.03234.x. 21226685 10.1111/j.1532-5415.2010.03234.x

[76] Xie L, Ma W, Du K, Huang Y, Li A, Wang H, et al. Association between exercise self-efficacy and physical activity in elderly individuals: a systematic review and meta-analysis. Front Psychol. 2025;16:1525277. 10.3389/fpsyg.2025.1525277. 40557371 10.3389/fpsyg.2025.1525277 PMC12185527

[77] Alberts L, Lyngs U, Lukoff K. Designing for sustained motivation: a review of self-determination theory in behaviour change technologies. Interact Comput. 2024:iwae040. 10.1093/iwc/iwae040.

[78] Goodarzi S, Teymouri Athar MM, Beiky M, Fathi H, Nakhaee Z, Omran SP, et al. Effect of physical activity for reducing anxiety symptoms in older adults: a meta-analysis of randomized controlled trials. BMC Sports Sci Med Rehabil. 2024;16(1):153. 10.1186/s13102-024-00947-w. 39014515 10.1186/s13102-024-00947-w PMC11251295

[79] Sciences NAo, Medicine B, Do, Sciences S, Division M, Behavioral B, et al. Social isolation and loneliness in older adults: opportunities for the health care system. National Academies; 2020. 10.17226/25663. 32510896

[80] Tsai HH, Cheng CY, Shieh WY, Chang YC. Effects of a smartphone-based videoconferencing program for older nursing home residents on depression, loneliness, and quality of life: a quasi-experimental study. BMC Geriatr. 2020;20(1):27. 10.1186/s12877-020-1426-2. 31992217 10.1186/s12877-020-1426-2 PMC6986028

[81] Stathi A, Greaves CJ, Thompson JL, Withall J, Ladlow P, Taylor G, et al. Effect of a physical activity and behaviour maintenance programme on functional mobility decline in older adults: the REACT (Retirement in Action) randomised controlled trial. Lancet Public Health. 2022;7(4):e316–26. 10.1016/s2468-2667(22)00004-4. 35325627 10.1016/S2468-2667(22)00004-4 PMC8967718

[82] Campbell E, Petermann-Rocha F, Welsh P, Celis-Morales C, Pell JP, Ho FK, et al. The effect of exercise on quality of life and activities of daily life in frail older adults: A systematic review of randomised control trials. Exp Gerontol. 2021;147:111287. 10.1016/j.exger.2021.111287. 33609689 10.1016/j.exger.2021.111287

[83] Racey M, Ali MU, Sherifali D, Fitzpatrick-Lewis D, Lewis R, Jovkovic M, et al. Effectiveness of nutrition interventions and combined nutrition and physical activity interventions in older adults with frailty or prefrailty: a systematic review and meta-analysis. Can Med Association Open Access J. 2021;9(3):E744–56.

