# Supplementary material for: Effectiveness of mobile health interventions on physical activity management in frail older adults: a systematic review and meta-analysis
Source: Eur Rev Aging Phys Act. 2026 Apr 11;23:16. doi: 10.1186/s11556-026-00411-3 (PMC13192223; doi:10.1186/s11556-026-00411-3)
Supplement: Supplementary file 1 — Supplementary Material 1. [file 11556_2026_411_MOESM1_ESM.docx]

**Supplementary Table 1.** Search terms and results for each database.

| **PubMed** | | |
| --- | --- | --- |
| 1# | | [Title/Abstract]  Telemedicine OR "Mobile?Health" OR "Health?Mobile" OR mHealth OR Telehealth OR eHealth OR "m?health" OR "e?health" OR telenurs* OR E-Mail OR Mails OR Email OR Emails OR "Electronic Mail" OR application OR App OR Portable OR Smartphone OR Smartwatch OR Phone OR Wearable OR Message OR Messaging OR Text OR Texting OR SMS OR "Short Message Service" OR Wearable OR Wireless OR Virtual OR "Artificial intelligenc*" OR "Mobile Devic*" OR Computer OR PDA |
| 2# | | [Title/Abstract]  Exercise OR Activity OR Activities OR Training OR Sport OR Athletic OR Sedentary OR Inactivity OR Walk OR walking OR Ambulation OR fitness OR running OR rope* OR Ambulat* |
| 3# | | [Title/Abstract]  “frail elderly”[Mesh] OR frailty[Title/Abstract] OR frail[Title/Abstract] OR frail*[Title/Abstract] OR “frailty syndrome”[Title/Abstract] OR “frail elder*”[Title/Abstract] |
| 4# | | "Randomized Controlled Trials as Topic"[Mesh] OR "Controlled Clinical Trials as Topic"[Mesh] OR "Randomized Controlled Trial"[Publication Type] OR "Controlled Clinical Trial"[Publication Type] OR random*[Title/Abstract] OR RCT[Title/Abstract] |
| 5# 242 results | | 1# AND 2# AND 3# AND 4# |
| **Web of Science** | | |
| 1# | | TS  Telemedicine OR "Mobile?Health" OR "Health?Mobile" OR mHealth OR Telehealth OR eHealth OR "m?health" OR "e?health" OR telenurs* OR E-Mail OR Mails OR Email OR Emails OR "Electronic Mail" OR application OR App OR Portable OR Smartphone OR Smartwatch OR Phone OR Wearable OR Message OR Messaging OR Text OR Texting OR SMS OR "Short Message Service" OR Wearable OR Wireless OR Virtual OR "Artificial intelligenc*" OR "Mobile Devic*" OR Computer OR PDA |
| 2# | | TS  Exercise OR Activity OR Activities OR Training OR Sport OR Athletic OR Sedentary OR Inactivity OR Walk OR walking OR Ambulation OR fitness OR running OR rope* OR Ambulat* |
| 3# | | TS  “frail elderly” OR frailty OR frail OR frail* OR “frailty syndrome” OR “frail elder*” |
| 4# | | TS  "Randomized Controlled Trial*" OR "Controlled Clinical Trial*" OR RCT* |
| 5# 495 results | | 1# AND 2# AND 3# AND 4# |
| **Cochrane Library** | | |
| 1# | | Record title  Telemedicine OR "Mobile Health" OR "Health Mobile" OR mHealth OR Telehealth OR eHealth OR (m?health) OR (e?health) OR telenurs* OR E-Mail OR Mails OR Email OR Emails OR "Electronic Mail" OR application OR App OR Portable OR Smartphone OR Smartwatch OR Phone OR Wearable OR Message OR Messaging OR Text OR Texting OR SMS OR "Short Message Service" OR Wearable OR Wireless OR Virtual OR (Artificial intelligenc*) OR (Mobile Devic*) OR Computer OR PDA |
| 2# | | Title Abstract Keywords  Exercise OR Activity OR Activities OR Training OR Sport OR Athletic OR Sedentary OR Inactivity OR Walk OR walking OR Ambulation OR fitness OR running OR rope* OR Ambulat* |
| 3# | | Title Abstract Keywords  (“frail elderly”) OR frailty OR frail OR frail* OR (“frailty syndrome”) OR (“frail elder*”) |
| 4 # 167 results | | 1# AND 2# AND 3# (Trials) |
| **EMBASE** | | |
| 1# | | ti, ab, kw  telemedicine OR 'mobile?health' OR 'health?mobile' OR 'mhealth' OR mhealth OR 'telehealth' OR telehealth OR 'ehealth' OR ehealth OR 'm?health' OR 'e?health' OR telenurs* OR 'e mail' OR 'e mail' OR mails OR 'email' OR email OR emails OR 'electronic mail' OR 'electronic mail' OR 'application' OR application OR 'app' OR app OR portable OR 'smartphone' OR smartphone OR 'smartwatch' OR smartwatch OR phone OR wearable OR message OR messaging OR text OR 'texting' OR texting OR sms OR 'short message service' OR 'short message service' OR wireless OR virtual OR 'artificial intelligenc*' OR 'mobile devic*' OR 'computer' OR computer OR 'PDA' |
| 2# | | ti, ab, kw  Exercise OR Activity OR Activities OR Training OR Sport OR Athletic OR Sedentary OR Inactivity OR Walk OR walking OR Ambulation OR fitness OR running OR rope* OR Ambulat* |
| 3# | | ti, ab, kw  “frail elderly” OR frailty OR frail OR frail* OR “frailty syndrome” OR “frail elder*” |
| 4# 424 results | | limitation: humans; Controlled Clinical Trial, Randomized Controlled Trial  1# AND 2# AND 3# |
| **CINAHL** | | |
| 1# | | 摘要关键词  Telemedicine OR "Mobile?Health" OR "Health?Mobile" OR mHealth OR Telehealth OR eHealth OR "m?health" OR "e?health" OR telenurs* OR E-Mail OR Mails OR Email OR Emails OR "Electronic Mail" OR application OR App OR Portable OR Smartphone OR Smartwatch OR Phone OR Wearable OR Message OR Messaging OR Text OR Texting OR SMS OR "Short Message Service" OR Wearable OR Wireless OR Virtual OR "Artificial intelligenc*" OR "Mobile Devic*" OR Computer OR PDA |
| 2# | | 摘要关键词  Exercise OR Activity OR Activities OR Training OR Sport OR Athletic OR Sedentary OR Inactivity OR Walk OR walking OR Ambulation OR fitness OR running OR rope* OR Ambulat* |
| 3# | | 摘要关键词  “frail elderly” OR frailty OR frail OR frail* OR “frailty syndrome” OR “frail elder*” |
| 4# | | 摘要关键词  "Randomized Controlled Trial*" OR "Controlled Clinical Trial*" OR RCT* |
| 5# 51 results | | 1# AND 2# AND 3# AND 4# |
| **Chinese National Knowledge Infrastructure (CNKI)** | | |
| 1# | | SU%（主题）=(‘应用程序’ + ‘APP’ + ‘穿戴设备’ + ‘智能设备’ + ‘移动医疗’ + ‘移动健康’ + ‘远程干预’ + ‘远程管理’ + ‘手环’ + ‘计步器’) |
| 2# | | TKA（篇关摘）=(‘手机’ + ‘电话’ + ‘短信’) |
| 3# | | TKA=(‘运动’ + ‘训练’ + ‘活动’ + ‘久坐’ + ‘步行’) |
| 4# | | TKA=(‘衰弱’ + ‘衰弱综合征’ + ‘衰弱症’ + ‘老年衰弱’) |
| 5# | | SU=(‘干预’ + ‘对照’ + ‘随机’ + ‘多中心’ + ‘试验’) |
| 6# 6 results | | (1# OR 2#) AND 3# AND 4# AND 5# |
| **SinoMed** | | |
| 1# | | [常用字段:智能] 应用程序 OR APP OR 穿戴设备 OR 智能设备 OR 移动医疗 OR 移动健康 OR 远程干预 OR 远程管理 OR 手机 OR 电话 OR 短信 OR 手环 OR 计步器 |
| 2# | | [常用字段:智能] 运动 OR 训练 OR 活动 OR 久坐 OR 步行 |
| 3# | | [常用字段:智能] 衰弱 OR 衰弱综合征 OR 衰弱症 OR 老年衰弱 |
| 4# | | [常用字段:智能] 干预 OR 对照 OR 随机 OR 多中心 OR 试验 |
| 5# 8 results | | 1# AND 2# AND 3# AND 4# |
| **WanFang Data** | | |
| 1# | | 主题:("应用程序" or "APP" or "穿戴设备" or "智能设备" or "移动医疗" or "移动健康" or "远程干预" or "远程管理" or "手环" or "计步器" or "手机" or "电话" or "短信") |
| 2# | | 主题:("运动" or "训练" or "活动" or "久坐" or "步行") |
| 3# | | 主题:("衰弱" or "衰弱综合征" or "衰弱症" or "老年衰弱") |
| 4# | | 主题:("干预" or "对照" or "随机" or "多中心" or "试验") |
| 5# 9 results | | limitation: 期刊论文、学位论文，主题词扩展  1# and 2# and 3# and 4# |
| **Cqvip** | | |
| 1# | R（摘要）=(应用程序 OR APP OR 穿戴设备 OR 智能设备 OR 移动医疗 OR 移动健康 OR 远程干预 OR 远程管理 OR 手机 OR 电话 OR 短信 OR 手环 OR 计步器) | |
| 2# | R=(运动 OR 训练 OR 活动 OR 久坐 OR 步行) | |
| 3# | R=(衰弱 OR 衰弱综合征 OR 衰弱症 OR 老年衰弱) | |
| 4# | M（题名或关键词）=(干预 OR 对照 OR 随机 OR 多中心 OR 试验) | |
| 5# 5 results | limitation: 期刊论文、学位论文  1# and 2# and 3# and 4# | |


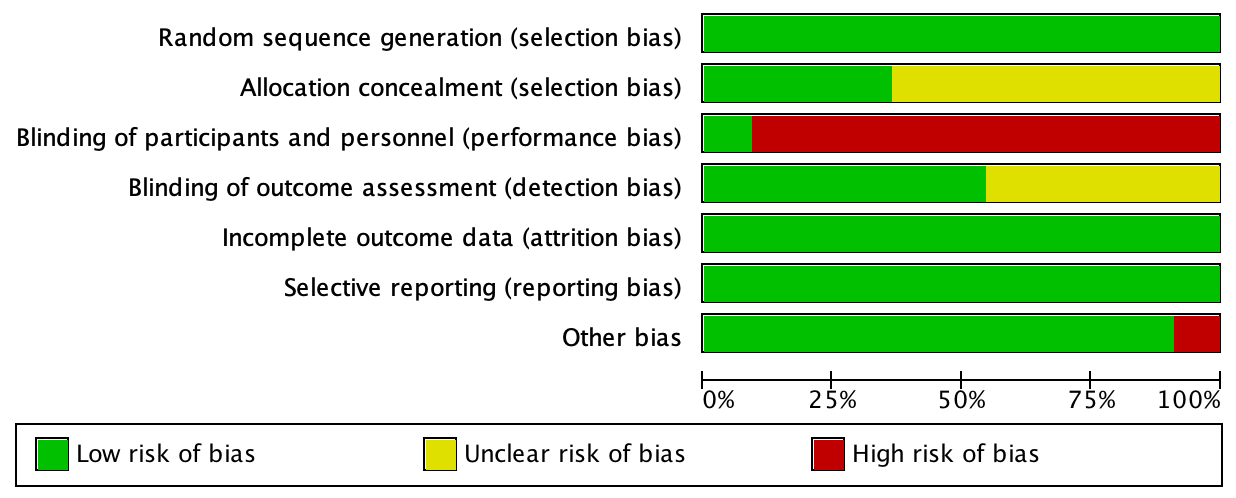


**Supplementary Fig. 1.** Risk of bias graph of RCTs.


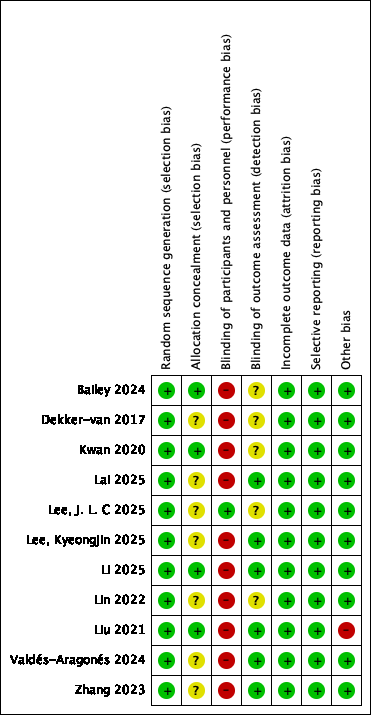


**Supplementary Fig. 2.** Risk of bias summary of RCTs.

**Supplementary Table 2.** Methodological quality evaluation of

quasi-experimental studies

| Quasi-experimental studies | Evaluation entry | | | | | | | | | Overall quality |
| --- | --- | --- | --- | --- | --- | --- | --- | --- | --- | --- |
|  | ① | ② | ③ | ④ | ⑤ | ⑥ | ⑦ | ⑧ | ⑨ |  |
| Li et al. (2025) | Yes | Yes | Yes | Yes | Yes | Yes | Yes | Yes | Yes | High |
| Xue et al. (2025) | Yes | Yes | Yes | Yes | Yes | Yes | Yes | Yes | Yes | High |
| Note: ① Is it clear in the study what is the ‘cause’ and what is the ‘effect’ (i.e., there is no confusion about which variable comes first)? ② Were the participants included in any comparisons similar? ③ Were the participants included in any comparisons receiving similar treatment/care, other than the exposure or intervention of interest? ④ Was there a control group? ⑤ Were there multiple measurements of the outcome both before and after the intervention/exposure? ⑥ Was follow-up complete, and if not, were differences between groups in terms of their follow-up adequately described and analyzed? ⑦ Were the outcomes of participants included in any comparisons measured in the same way? ⑧ Were outcomes measured in a reliable way? ⑨ Was appropriate statistical analysis used? | | | | | | | | | | |

**Supplementary Table 3.** Implementation Outcomes.

| Author | Reach | Feasibility | Adherence | Safety (N=AE) |
| --- | --- | --- | --- | --- |
| Bailey et al. (2024) | 72% | 83% | NR | 0 |
| Dekker-van et al. (2017) | 18% | 97% | 68% | NR |
| Kwan et al. (2020) | 63% | 91% | 97% | NR |
| Lai et al. (2025) | 100% | 100% | NR | NR |
| Lee, J. L. C et al. (2025) | 84% | 89% | Workshop attendance: 93% Outdoor self-practice attendance: 71% App usage: 69% average：78% | 2 (1 case of a fall, and 1 case of knee and back pain) |
| Li, N et al. (2025) | 92% | 79% | 61% | 3(1 case of knee pain, a case og foot pain, and a case of transient dizziness. All symptoms were effectively managed with rest) |
| Lin et al. (2022) | NR | 100% | NR | NR |
| Liu et al. (2021) | 93% | 100% | Device wearing: 94% (during the intervention period) / 92% (during the follow-up period); Exercise: 85%  average：90% | 0 |
| Piau et al. (2021) | NR | 74% | 100% (in the first month of intervention) 83% (in the following 2 months) 94% (in the following 3 months) average：92% | 2(1 case of a fall and 1 case of a fracture, not attributable to the intervention) |
| Valdés-Aragonés et al. (2024) | 61% | 87% | NR | The intervention group demonstrated a significantly lower risk of experiencing falls compared to the control group over the 6-month follow-up period (Beta = −0.29, 95% CI: −0.53 to −0.04; P = 0.025) |
| Lee, Kyeongjin (2025) | 100% | 96% | NR | NR |
| Zhang et al. (2023) | NR | 95% | 76% | 0 |
| Li, P. S et al. (2025) | 98% | 100% | NR | 0 |
| Xue et al. (2025) | 51% | 88% | 84% | 0 |
| Notes: AE, adverse events. | | | | |
